# The role of lifestyle in the association between long-term ambient air pollution exposure and cardiovascular disease: a national cohort study in China

**DOI:** 10.1186/s12916-024-03316-z

**Published:** 2024-03-05

**Authors:** Xiangming Hu, Luke D. Knibbs, Yingling Zhou, Yanqiu Ou, Guang-Hui Dong, Haojian Dong

**Affiliations:** 1 Department of Cardiology, Guangdong Cardiovascular Institute, Guangdong Provincial People’s Hospital (Guangdong Academy of Medical Sciences), Southern Medical University, Guangzhou, 510080 China; 2 School of Public Health, The University of Sydney, Camperdown, NSW 2006 Australia; 3 Public Health Research Analytics and Methods for Evidence, Public Health Unit, Sydney Local Health District, Camperdown, NSW 2050 Australia; 4 Guangdong Provincial Engineering Technology Research Center of Environmental Pollution and Health Risk Assessment, Department of Occupational and Environmental Health, School of Public Health, Sun Yat-Sen University, Guangzhou, 510080 China

**Keywords:** Ambient air pollution, Cardiovascular disease, Healthy lifestyle, China Health and Retirement Longitudinal Study

## Abstract

**Background:**

Cardiovascular disease (CVD) caused by air pollution poses a considerable burden on public health. We aim to examine whether lifestyle factors mediate the associations of air pollutant exposure with the risk of CVD and the extent of the interaction between lifestyles and air pollutant exposure regarding CVD outcomes.

**Methods:**

We included 7000 participants in 2011–2012 and followed up until 2018. The lifestyle evaluation consists of six factors as proxies, including blood pressure, blood glucose, blood lipids, body mass index, tobacco exposure, and physical activity, and the participants were categorized into three lifestyle groups according to the number of ideal factors (unfavorable, 0–1; intermediate, 2–4; and favorable, 5–6). Satellite-based spatiotemporal models were used to estimate exposure to ambient air pollutants (including particles with diameters ≤ 1.0 μm [PM_1_], ≤ 2.5 μm [PM_2.5_], ≤ 10 μm [PM_10_], nitrogen dioxide [NO_2_], and ozone [O_3_]). Cox regression models were used to examine the associations between air pollutant exposure, lifestyles and the risk of CVD. The mediation and modification effects of lifestyle categories on the association between air pollutant exposure and CVD were analyzed.

**Results:**

After adjusting for covariates, per 10 μg/m^3^ increase in exposure to PM_1_ (HR: 1.09, 95% CI: 1.05–1.14), PM_2.5_ (HR: 1.04, 95% CI: 1.00–1.08), PM_10_ (HR: 1.05, 95% CI: 1.03–1.08), and NO_2_ (HR: 1.11, 95% CI: 1.05–1.18) was associated with an increased risk of CVD. Adherence to a healthy lifestyle was associated with a reduced risk of CVD compared to an unfavorable lifestyle (HR: 0.65, 95% CI: 0.56–0.76 for intermediate lifestyle and HR: 0.41, 95% CI: 0.32–0.53 for favorable lifestyle). Lifestyle played a significant partial mediating role in the contribution of air pollutant exposure to CVD, with the mediation proportion ranging from 7.4% for PM_10_ to 14.3% for PM_2.5_. Compared to an unfavorable lifestyle, the relative excess risk due to interaction for a healthier lifestyle to reduce the effect on CVD risk was − 0.98 (− 1.52 to − 0.44) for PM_1_, − 0.60 (− 1.05 to − 0.14) for PM_2.5_, − 1.84 (− 2.59 to − 1.09) for PM_10_, − 1.44 (− 2.10 to − 0.79) for NO_2,_ and − 0.60 (− 1.08, − 0.12) for O_3_.

**Conclusions:**

Lifestyle partially mediated the association of air pollution with CVD, and adherence to a healthy lifestyle could protect middle-aged and elderly people from the adverse effects of air pollution regarding CVD.

**Supplementary Information:**

The online version contains supplementary material available at 10.1186/s12916-024-03316-z.

## Background

Cardiovascular disease (CVD) is the leading cause of mortality worldwide [1]. In the updated 2019 Global Burden of Disease, the number of patients with CVD in 2019 reached 55.4 million worldwide, an increase of 77.12% compared to 1990, with the largest increases occurring in South and East Asia [1]. Therefore, considering the poor prognosis, attention should be given to the management of risk factors for CVD.

Ambient air pollution, as the most important environmental risk factor for health globally, is a major cause of CVD and CVD-related mortality [2]. Particulate matter (PM) (including PM ≤ 10 μm [PM_10_], PM ≤ 2.5 μm [PM_2.5_], which also includes PM ≤ 1 μm [PM_1_]), nitrogen dioxide (NO_2_), and ozone (O_3_) are well-documented outdoor air pollutants in studies of CVD [3–6]. Curbing the disease burden related to air pollution exposure is a long-term endeavor. In addition to encouraging the control of emissions of major air pollutants at their source, modifiable individual lifestyle factors are also important [7].

Accumulating evidence has shown that unhealthy lifestyle factors, i.e., physiological and behavioral risk factors, contribute to a large burden of CVD [7, 8]. Conversely, adherence to a healthy lifestyle for CVD prevention may reduce the adverse effects of air pollution exposure [9–12]. Although studies have investigated the association of lifestyle factors between air pollution exposure and CVD risk, numerous other unresolved questions remain [8–12]. First, despite lifestyle factors being interrelated, few studies have established a composite lifestyle assessment to reflect and assess its impact on the adverse effect of ambient air pollution exposure. In addition, the extent to which adherence to a healthy lifestyle modifies and mediates the link between ambient air pollution exposure and CVD incidence is still unknown. Third, it is unclear whether these effects are consistent across different levels of healthy lifestyles, different types of ambient air pollutants, and different levels of air pollution exposure. More importantly, evidence for protective measures against CVD in the context of high levels of air pollution is still lacking.

Hence, a study that evaluates the associations between air pollutant exposure, lifestyles and the risk of CVD is crucial to develop targeted strategies for the control of CVD. We used a large national database from China to determine the effect of a healthy lifestyle on the association between ambient air pollution exposure and CVD.

## Methods

### Study population

The China Health and Retirement Longitudinal Study (CHARLS) is an ongoing national longitudinal study conducted by Peking University that began in 2011. In short, the CHARLS employed a probability proportionated to size sampling, covering more than 17,000 representative respondents aged 45 years and above from 28 provinces across China, with a response rate of about 85% (Additional file 1: Fig. S1), as detailed elsewhere [13]. In a face-to-face setting, high-quality data were collected via standardized questionnaires about demographic information, health conditions, and medical history. The participants underwent a physical examination and blood biomarker detection. All participants were followed up every 2 years after the baseline survey.

**Fig. 1:**
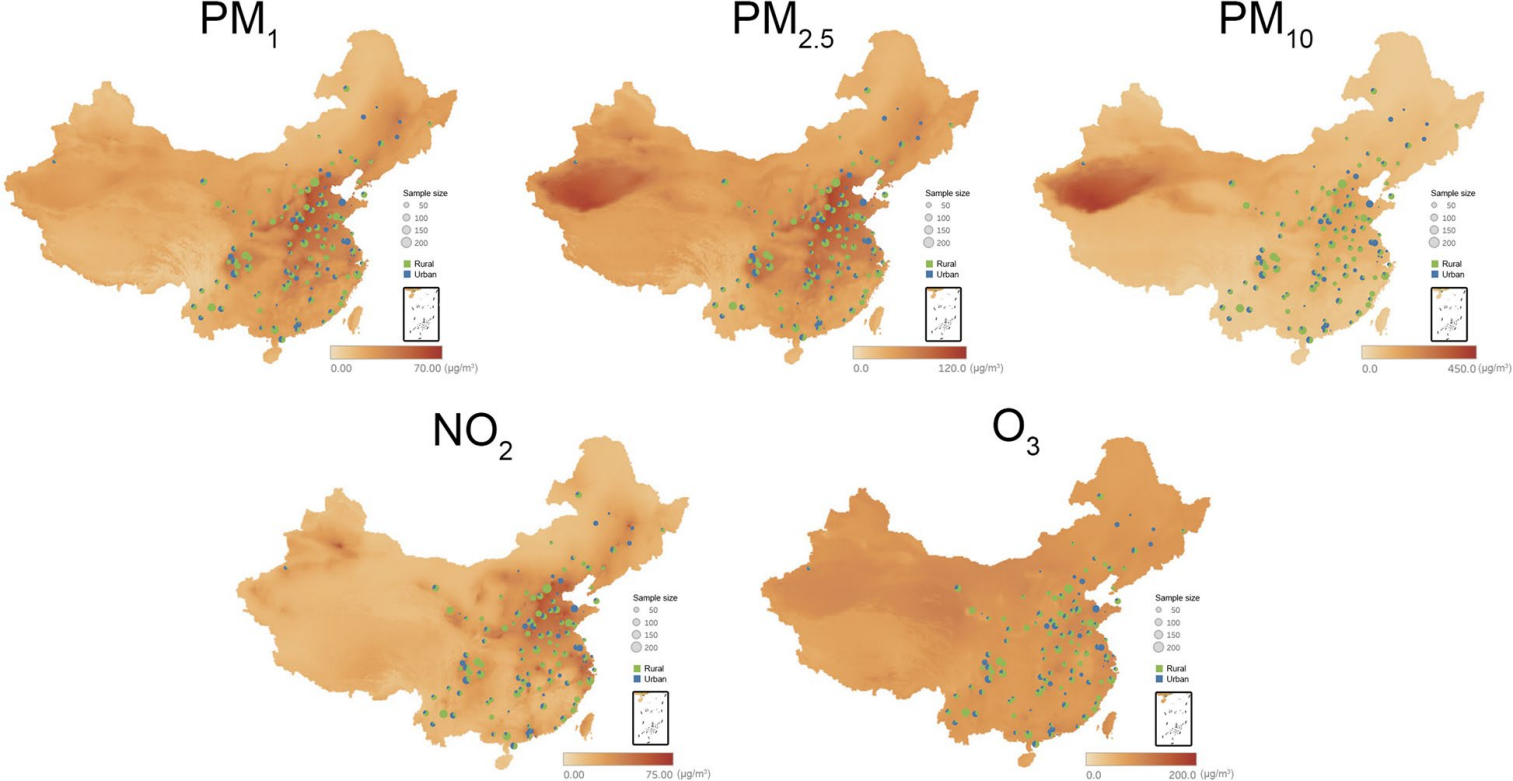
Maps of the distribution of studied ambient air pollutants and enrolled participant locations across China. NO_2_, nitrogen dioxide; O_3_, ozone; PM_1_, particulate matter with an aerodynamic diameter less than 1 μm; PM_2.5_, particulate matter with an aerodynamic diameter less than 2.5 μm; PM_10_, particulate matter with an aerodynamic diameter < 10 μm

In this study, we extracted data from the CHARLS from Wave 2011 to Wave 2018, with the following inclusion criteria: (1) individuals aged ≥ 45 years; (2) individuals with sufficient information to measure healthy lifestyle score; and (3) individuals without CVD history before Wave 2011. The exclusion criteria were as follows: (1) individuals missing information about baseline CVD and (2) individuals who died or were lost to follow-up. Among them, 474 participants aged less than 45 years, 8047 participants without information to measure healthy lifestyle score, 1306 participants with CVD history or missing baseline CVD information, 605 participants who died, and 273 participants who were lost to follow-up were excluded. Eventually, the study included 7000 participants (Additional file 1: Fig. S2). Figure 1 describes the distribution of the study sites with the background map of air pollutants across China. The ethical committee of Peking University approved the CHARLS (IRB00001052-11015). All participants have submitted a written informed consent.

### Ambient air pollution exposure acquisition

Details of the measurement of ambient air pollution exposure are presented in the appendix (Additional file 1: Method S1) [14–23]. Briefly, daily concentrations of PM_1_ and PM_2.5_ were estimated at a high spatial resolution (0.1° × 0.1°) by satellite-based spatiotemporal models. The concentrations of PM_10_, NO_2_, and O_3_ were derived from appropriately located air monitors in the district where each study participant lived. Geographic matching was performed based on the residence where each enrolled participant lived, accurate to urban or rural level (as defined by the 2013 urban and rural statistical division codes of the National Bureau of Statistics of China). We geocoded the addresses where each participant’s residence was located and superimposed a grid to predict monthly ambient air pollutant concentrations. We then averaged the ambient air pollutant (PM_1_, PM_2.5_, PM_10_, NO_2_, and O_3_) concentrations for each participant over the 1-year period before the interview day to assign exposure. Other exposure averaging periods were assessed, as outlined in the Sensitivity analyses section that follows.

### Healthy lifestyle measurement

Based on the Life’s Simple 7 proposed by the American Heart Association and previous studies [9, 24, 25], we constructed a healthy lifestyle score to generalize modifiable risk factors, including blood pressure, blood glucose, blood lipids, body mass index (BMI), smoking, and physical activity. Blood pressures were taken as the mean of three measurements at 45-s intervals recorded on the right upper arm after 5 min of sitting rest using an OmronTM HEM-7112 Blood Pressure Monitor by trained staff. Fasting blood glucose and lipid profiles were measured by enzymatic colorimetric tests. Height and weight were measured with light clothing and without shoes to calculate BMI. Smoking status was categorized as either current smoking or not. Ideal physical activity was defined as 30 min of vigorous exercise (including heavy lifting, digging, ploughing, aerobics, fast bicycling, and cycling with a heavy load) or moderate physical activity (including carrying light loads, bicycling at a regular pace, or mopping the floor) at least three times a week. We did not include dietary factors since the CHARLS did not collect specific dietary information. The correlation between the various lifestyle factors is shown in Additional file 1: Fig. S3. Each of these six factors was assigned a score of 0 to 1 (Additional file 1: Table S1), and we calculated the total score for the modifiable lifestyle factors on a scale from 0 to 6, with higher scores indicating greater adherence to a healthy lifestyle. Participants were divided into three categories of lifestyle according to the scores (unfavorable, 0–1 point; intermediate, 2–4 points; favorable, 5–6 points) [12]. The proportions of ideal factors in different lifestyle groups are shown in Additional file 1: Fig. S4.

### Cardiovascular disease definition

CVD was defined by medical diagnosis as reported in response to the following questionnaire items: “Have you ever been diagnosed with heart attack, coronary heart disease, angina, congestive heart failure, other heart problems by a doctor since the last interview?”; “Have you ever been diagnosed with stroke by a doctor since the last interview?” [26]. Participants were considered to have CVD if they gave a positive response to at least one question.

### Covariates

Data for age (years), sex (male/female), education (primary school and below/junior high school/high school and above), residence (rural/urban), per capita household annual income (¥, hereafter referred to as income), and alcohol consumption (drink more than once a month/drink less than once a month/never drink) were obtained from standard questionnaires.

### Blood test

Participants were requested to fast for 8–12 h before blood sample collection. Blood samples were stored at – 70 °C, and bioassays were performed at uniform quality control by the National Centers for Disease Control. Blood lipids (high-density lipoprotein cholesterol, low-density lipoprotein cholesterol, total cholesterol, triglycerides) and blood glucose were measured by enzymatic colorimetric tests. Hypersensitive C-reactive protein was detected by immunoturbidimetric assay.

### Statistical analyses

Participants were divided into three groups according to the extent of healthy lifestyle as mentioned above. *P* values for trends were calculated with each type of healthy lifestyle taken as a unit by using linear regression analyses and the Wald chi-square test for trend analysis of continuous and categorical variables across the three groups. We developed a directed acyclic graph (DAG) to guide covariate selection (Additional file 1: Fig. S5). A Cox regression model was used to examine the associations between air pollutant exposure (per 10 μg/m^3^ increase) and CVD and between lifestyles and CVD (expressed as hazard ratio [HR] and 95% confidence interval [CI]). The results of the proportional hazards assumption in all models were satisfactory. Participants were divided into five groups based on the levels of different air pollutant exposure, with quintile 1 (Q1) representing low level exposure and Q2–Q5 representing high level exposure to air pollution. Stratification analysis was conducted to examine the effect of lifestyle and CVD in different air pollutant exposure (low: Q1 and high: Q2–Q5) subgroups. Potential confounders that were significant in the baseline comparison or considered clinically important were selected for the multivariate adjusted models. Two main models were employed for clinically significant covariate adjustment: model 1 (crude model) was unadjusted, and model 2 (maximally adjusted model) was adjusted for age, sex, education level, residence, alcohol consumption, and income.

To fully identify the modification effect of a healthy lifestyle on the association between air pollutant exposure and CVD, we employed three methods. First, the modification on a multiplicative scale (using HR for the term between the healthy lifestyle category [unfavorable, intermediate, favorable] and air pollutant exposure) was tested using the likelihood ratio test, with statistical significance indicated by a confidence interval of HR that did not include 1. Second, the marginal effects using the means were calculated in the dimension of healthy lifestyle categories and the dimension of different air pollutant concentrations. Third, we assessed the modification effect on an additive scale (using HR for the term between the lifestyle categories and air pollutant exposure) by calculating the relative excess risk due to interaction (RERI), and the additive interaction was considered statistically significant when its confidence interval did not include 0. Participants were divided into two categories of lifestyle (unfavorable: 0–1 scores and intermediate and favorable: 2–6 scores) for the potential additive interaction analysis.

Given that there are associations of air pollutant exposure and healthy lifestyle with incident CVD (Additional file 1: Fig. S5), we investigated whether the association between air pollution exposure and incident CVD was mediated by different lifestyle categories using the “mediation” R package with 10,000 bootstraps.

Several sensitivity analyses were then performed to test the robustness of the studied association. First, the effects of adherence to a single lifestyle factor on CVD at different levels of air pollutant exposure were identified. Second, subgroup analyses (age and sex) of the effect of a healthy lifestyle on the risk of CVD in different air pollutant exposures were explored. Then, we developed a series of models to verify the robustness of the main results as follows: (1) as the prevalence of depressive symptoms evaluated by a 10-question version of the Center for Epidemiologic Studies-Depression (CES-D) scale is considered a novel CVD risk factor, we removed participants without CES-D scores (*n* = 6881) and repeated the main analysis with further adjustment for depressive symptoms. Depressive symptoms were defined by a CES-D score of ≥ 12 [27]; (2) to account for the impact of household solid fuel use on the main outcome, we developed a new model that accounts for household solid fuel use (*n* = 6947). Domestic solid fuel use (coal, crop residue or wood burning) for cooking (yes/no) were obtained from standard questionnaires; (3) due to possible measurement bias associated with dynamic changes in air pollutant exposure during the follow-up period, we used a time-varying model to calculate averaged cumulative air pollutant exposure from the year prior to the baseline visit to the year of outcome instead of 1-year exposure before enrolment; (4) to check the representativeness of the 1-year exposure analysis, a 3-year average air pollution exposure before baseline survey was calculated; (5) as sleep is included by the AHA as a new component of Life’s Essential 8, we included nighttime sleep duration in the lifestyle score and reclassified participants into three new lifestyle categories (unfavorable, 0–1 point; intermediate, 2–4 points; favorable, 5–7 points) (*n* = 6951). According to a previous meta-analysis on the association between sleep duration and cardiovascular outcomes, sleep disorder was defined by a self-reported night sleep duration of < 6 h or > 8 h [28]; (6) each of these six factors was assigned a score of 0 to 2 (Additional file 1: Table S1) instead of 0 to 1, and participants were divided into three lifestyle categories according to their lifestyle scores (unfavorable, 0–5 points; intermediate, 6–9 points; favorable, 10–12 points); (7) we replicated the main analysis by excluding patients without covariates (*n* = 6235) or who changed their residence (*n* = 5519); (8) since lung function could affect the effects of air pollution on disease, we performed the analysis with inclusion restricted to participants without chronic lung disease in all the above explored correlations (*n* = 5689). A history of chronic lung disease was self-reported; (9) considering participants who died during the follow-up period, a competing risk model was employed; and (10) we replaced the death by free of CVD and repeated the analyses (*n* = 7605). The method of the last observation carried forward or the means and medians were used to interpolate the missing data. *P* value < 0.05 (two-sided) was considered statistical significance. The Stata (StataCorp LLC, version 15.0) and R software (version 4.2.2) were used for the data analyses.

## Results

### Baseline characteristics

Table 1 shows the baseline characteristics of the participants from the CHARLS. Among 7000 participants, the mean age (standard deviation) was 58.4 (8.8) years, and males accounted for 46.4%. Totally, 818 participants (11.7%) had unfavorable lifestyles, 5308 participants (75.8%) had intermediate lifestyles, and 874 participants (12.5%) had favorable lifestyles. Adults with favorable lifestyle were more likely to be younger, female, less educated, and reside in rural areas. Alcohol consumption and hyperlipemia were more prevalent among adults with unfavorable lifestyle. The hs-CRP levels were the lowest in the healthy lifestyle group. The concentrations of ambient air pollutant exposure were slightly higher in populations with unfavorable lifestyle than in those with favorable lifestyle (more detail provided in Additional file 1: Table S2). In the group exposed to low levels (Q1) of air pollution, the average exposure levels of PM_1_, PM_2.5_, PM_10_, NO_2_, and O_3_ were observed to be 20.25 ± 3.92, 29.31 ± 6.04, 53.81 ± 9.37, 15.72 ± 2.48, and 86.14 ± 2.96 μg/m^3^, respectively (Additional file 1: Table S3). The most pronounced difference among the three lifestyle groups was observed in the proportion of individuals with target blood pressure, while the smallest difference was in the proportion of nonsmokers. In the unfavorable lifestyle group, most people (84%) had one ideal lifestyle factor, which was predominantly nonsmoking. In the intermediate lifestyle group, nearly 70% of people had three or more ideal lifestyle factors, predominantly characterized by target total cholesterol, non-overweight, and nonsmoking. In the favorable lifestyle group, the proportion of each ideal lifestyle factor exceeded 65%.

**Table 1:** Baseline characteristic of the enrolled participants according to different lifestyle categories

	Total *N* = 7000	Unfavorable lifestyle *N* = 818	Intermediate lifestyle *N* = 5308	Favorable lifestyle *N* = 874	*P* value
Age, years	58.43 ± 8.80	58.87 ± 8.27	58.79 ± 8.90	55.85 ± 8.16	< 0.001
Male sex	3247 (46.39%)	511 (62.47%)	2484 (46.80%)	252 (28.83%)	< 0.001
Education^a^					< 0.001
Primary school and below	5647 (70.47%)	518 (63.33%)	3700 (69.72%)	641 (73.34%)	
Junior high school	1592 (19.87%)	200 (24.45%)	1087 (20.48%)	141 (16.13%)	
High school and above	774 (9.66%)	100 (12.22%)	520 (9.80%)	92 (10.53%)	
Residence					< 0.001
Rural	4662 (66.60%)	467 (57.09%)	3580 (67.45%)	615 (70.37%)	
Urban	2338 (33.40%)	351 (42.91%)	1728 (32.55%)	259 (29.63%)	
Per capita household annual income^a^, RMB	5513.33 (1899.38–13,500.00)	6867.67 (2333.33–15,333.33)	5245.71 (1815.83–13,265.00)	5920.00 (2056.25–13,500.00)	< 0.001
Smoking	2678 (38.26%)	540 (66.01%)	2020 (38.06%)	118 (13.50%)	< 0.001
Alcohol consumption					< 0.001
Drink more than once a month	1829 (26.13%)	296 (36.19%)	1377 (25.94%)	156 (17.85%)	
Drink less than once a month	575 (8.21%)	62 (7.58%)	440 (8.29%)	73 (8.35%)	
Never drink	4596 (65.66%)	460 (56.23%)	3491 (65.77%)	645 (73.80%)	
Physical activity	2016 (28.80%)	33 (4.03%)	1389 (26.17%)	594 (67.96%)	< 0.001
Nighttime sleep duration^a^	6.39 ± 1.89	6.40 ± 1.86	6.39 ± 1.90	6.39 ± 1.85	0.892
Solid fuel use for cooking^a^	4012 (57.75%)	407 (50.12%)	3095 (58.73%)	510 (58.96%)	< 0.001
CES-D score^a^	8.04 ± 6.12	7.11 ± 5.82	8.04 ± 6.09	8.95 ± 6.42	< 0.001
BMI^a^, kg/m^2^	23.49 ± 3.81	26.48 ± 3.82	23.32 ± 3.73	21.75 ± 2.59	< 0.001
SBP, mmHg	128.32 ± 20.84	141.60 ± 18.29	128.96 ± 20.50	112.01 ± 13.37	< 0.001
DBP, mmHg	74.98 ± 12.00	82.53 ± 11.12	75.22 ± 11.65	66.40 ± 9.35	< 0.001
TC, mg/dL	194.01 ± 38.40	226.35 ± 40.33	192.59 ± 36.77	172.39 ± 24.96	< 0.001
Triglycerides, mg/dL	104.43 (74.34–152.22)	147.79 (102.66–230.10)	103.55 (74.34–148.68)	83.19 (62.84–114.17)	< 0.001
LDL-C, mg/dL	116.76 ± 34.53	137.77 ± 40.05	115.98 ± 33.73	101.93 ± 22.56	< 0.001
HDL-C, mg/dL	51.48 ± 15.32	47.31 ± 15.78	51.80 ± 15.40	53.46 ± 13.60	< 0.001
Blood glucose, mg/dL	109.64 ± 34.94	128.59 ± 47.92	109.27 ± 33.61	94.16 ± 13.87	< 0.001
Hs-CRP, mg/dL	0.98 (0.53–2.02)	1.46 (0.77–2.88)	0.97 (0.54–1.99)	0.67 (0.41–1.31)	< 0.001
Chronic lung disease^a^	595 (8.51%)	68 (8.31%)	463 (8.73%)	64 (7.33%)	0.449
Lipids lowering therapy	243 (3.47%)	60 (7.33%)	166 (3.13%)	17 (1.95%)	< 0.001
Ambient air pollutants					
PM_1_, μg/m^3^	39.97 ± 13.80	42.01 ± 13.64	39.87 ± 13.82	38.69 ± 13.64	< 0.001
PM_10_, μg/m^3^	93.44 ± 28.05	97.49 ± 27.98	93.32 ± 27.99	90.33 ± 28.05	< 0.001
PM_2.5_, μg/m^3^	52.61 ± 15.86	54.57 ± 15.61	52.61 ± 15.82	50.84 ± 16.08	< 0.001
NO_2_, μg/m^3^	29.23 ± 10.79	31.15 ± 10.74	29.20 ± 10.76	27.57 ± 10.74	< 0.001
O_3_, μg/m^3^	95.33 ± 6.49	95.77 ± 7.05	95.26 ± 6.46	95.30 ± 6.11	0.145

Data are shown as means ± standard deviations or numbers (percentages)

*Abbreviations*: *BMI* Body mass index, *CES-D* Center for epidemiologic studies-depression, *CVD* Cardiovascular disease, *DBP* Diastolic blood pressure, *HDL-C* High-density lipoprotein cholesterol, *LDL-C* Low-density lipoprotein cholesterol, *NO*_*2*_ Nitrogen dioxide, *O*_*3*_ Ozone, *PM*_*1*_ Particulate matter with an aerodynamic diameter less than 1 μm, *PM*_*2.5*_ Particulate matter with an aerodynamic diameter less than 2.5 μm, *PM*_*10*_ Particulate matter with an aerodynamic diameter < 10 μm, *SBP* Systolic blood pressure, *TC* Total cholesterol

^a^Missing data: 11 for BMI, one for education, 764 for income, six for chronic lung disease, 56 for nighttime sleep duration, 444 for CES-D score, and 65 for solid fuel use

### Associations of air pollution exposure and lifestyle with incident CVD

**Fig. 2:**
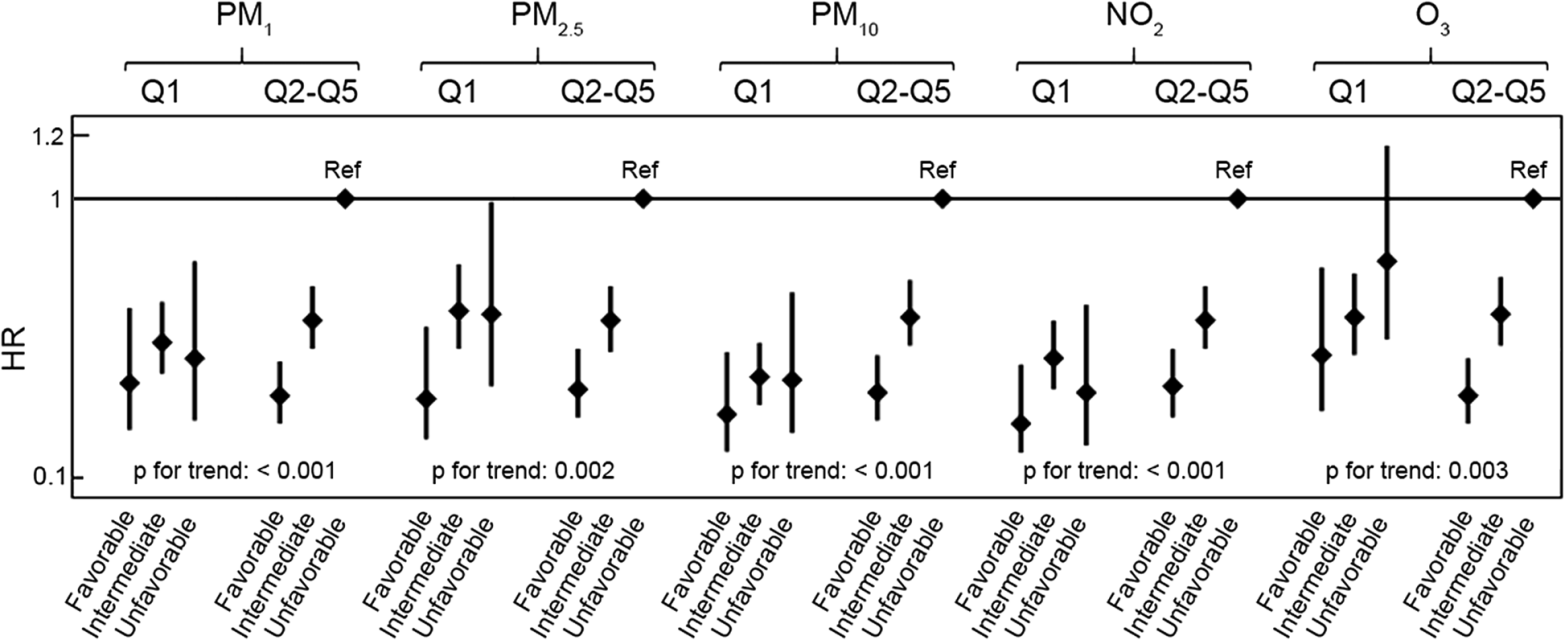
Joint effects of lifestyle and air pollutant exposure on the incidence of CVD. Unfavorable lifestyle as reference. Model adjusted for age, sex, education, residence, alcohol consumption, and income. CI, confidence interval; CVD, cardiovascular disease; HR, hazard ratio; NO_2_, nitrogen dioxide; O_3_, ozone; PM_1_, particulate matter with an aerodynamic diameter less than 1 μm; PM_2.5_, particulate matter with an aerodynamic diameter less than 2.5 μm; PM_10_, particulate matter with an aerodynamic diameter < 10 μm

With a median follow-up of 7 years, 1187 participants developed CVD. As shown in Table 2, after adjusting for covariates, per 10 μg/m^3^ increase in exposure to PM_1_ (HR: 1.09, 95% CI: 1.05–1.14), PM_2.5_ (HR: 1.04, 95% CI: 1.00–1.08), PM_10_ (HR: 1.05, 95% CI: 1.03–1.08), and NO_2_ (HR: 1.11, 95% CI: 1.05–1.18) was associated with an increased risk of CVD, while adherence to a healthy lifestyle was associated with a reduced risk of CVD (HR: 0.65, 95% CI: 0.56–0.76 for intermediate lifestyle and HR: 0.41, 95% CI: 0.32–0.53 for favorable lifestyle, Additional file 1: Table S4). Figure 2 shows the joint effects between lifestyle and different air pollutant exposure categories (Q1 vs. Q2–Q5) on the incidence of CVD. At high levels of air pollution exposure, an improved lifestyle had greater cardiovascular benefits compared to a poor lifestyle, with approximately 38% and 62% decreased risks of CVD in intermediate and favorable lifestyles, respectively (Additional file 1: Table S5). In the case of low levels of air pollution exposure, adherence to a favorable lifestyle minimalized the risks of CVD (Additional file 1: Table S5). The effect of a single lifestyle factor on the risk of CVD was significant at higher levels of air pollution exposure (Q2–Q5), except that target blood pressure levels were more beneficial at lower air pollution exposure (Additional file 1: Table S6).

**Table 2:** The HR (95% CI) of the associations between air pollution (per 10 μg/m^3^ increase) and CVD with and without adjustment for lifestyle

	Unadjusted for lifestyle	Adjusted for lifestyle^a^
PM_1_	1.09 (1.05–1.14)	1.08 (1.04–1.13)
PM_2.5_	1.04 (1.00–1.08)	1.04 (1.00–1.07)
PM_10_	1.05 (1.03–1.08)	1.05 (1.03–1.07)
NO_2_	1.11 (1.05–1.18)	1.10 (1.04–1.16)
O_3_	1.04 (0.95–1.14)	1.03 (0.94–1.13)

*CI* Confidence interval, *CVD* Cardiovascular disease, *HR* Hazard ratio, *NO*_*2*_ Nitrogen dioxide, *O*_*3*_ Ozone, *PM*_*1*_ Particulate matter with an aerodynamic diameter less than 1 μm, *PM*_*2.5*_ Particulate matter with an aerodynamic diameter less than 2.5 μm, *PM*_*10*_ Particulate matter with an aerodynamic diameter < 10 μm

^a^Model adjusted for age, sex, education, residence, alcohol consumption, and income

### Mediation analysis of lifestyle in the association between air pollution exposure and incident CVD

Table 3 shows the HRs (95% CIs) from crude and adjusted models for air pollutant exposure and incidence of CVD stratified by different lifestyle groups. Among participants with unfavorable lifestyle, in the full-adjusted model, a 10 μg/m^3^ increase in PM_1_, PM_2.5_, PM_10_, and NO_2_ was significantly associated with a 17% (HR: 1.17, 95% CI: 1.05–1.31), 13% (HR: 1.13, 95% CI: 1.02–1.24), 9% (HR: 1.09, 95% CI: 1.04–1.15), and 25% (HR: 1.25, 95% CI: 1.09–1.44) higher incidence for CVD prevalence, respectively. However, the association of PM_1_, PM_2.5_, PM_10_, and NO_2_ with CVD was not significant in the favorable lifestyle group. The effect of O_3_ on incident CVD was not significant among the three groups. Lifestyle plays a significant mediating role in the contribution of air pollutant exposure to CVD, with the proportion of mediated effects of 8.0% (95% CI: 3.6%–17.8%) in PM_1_, 14.3% (95% CI: 5.6%–54.1%) in PM_2.5_, 7.4% (95% CI: 3.8%–13.3%) in PM_10_, and 12.0% (95% CI: 6.3%–24.6%) in NO_2_.

**Table 3:** The association between air pollutants exposure (per 10 μg/m^3^) and incident CVD in different lifestyle categories, and the mediation effect of lifestyle categories to air pollution in CVD

	Unfavorable HR (95% CI)	Intermediate HR (95% CI)	Favorable HR (95% CI)	Mediation proportion (%) (95% CI)
PM_1_				8.0 (3.6–17.8)
Model 1	1.15 (1.04–1.28)	1.09 (1.04–1.14)	0.97 (0.84–1.13)	
*P* value	0.008	0.001	0.697	
Model 2	1.17 (1.05–1.31)	1.08 (1.03–1.13)	0.97 (0.83–1.14)	
*P* value	0.005	0.003	0.762	
PM_2.5_				14.3 (5.6–54.1)
Model 1	1.12 (1.02–1.23)	1.04 (1.00–1.09)	0.97 (0.85–1.10)	
*P* value	0.020	0.058	0.591	
Model 2	1.13 (1.02–1.24)	1.03 (0.98–1.07)	0.96 (0.84–1.10)	
*P* value	0.016	0.238	0.552	
PM_10_				7.4 (3.8–13.3)
Model 1	1.09 (1.03–1.14)	1.05 (1.02–1.07)	1.01 (0.94–1.08)	
*P* value	0.001	< 0.001	0.798	
Model 2	1.09 (1.04–1.15)	1.04 (1.02–1.07)	1.01 (0.94–1.09)	
*P* value	0.001	< 0.001	0.751	
NO_2_				12.0 (6.3–24.6)
Model 1	1.21 (1.06–1.38)	1.09 (1.03–1.16)	0.97 (0.80–1.17)	
*P* value	0.004	0.005	0.751	
Model 2	1.25 (1.09–1.44)	1.08 (1.01–1.15)	0.98 (0.80–1.20)	
*P* value	0.001	0.017	0.819	
O_3_				-
Model 1	1.21 (0.98–1.50)	1.03 (0.93–1.14)	0.83 (0.59–1.16)	
*P* value	0.056	0.920	0.419	
Model 2	1.24 (0.99–1.54)	1.00 (0.90–1.11)	0.87 (0.62–1.22)	
*P* value	0.071	0.556	0.279	

Model 1: crude model

Model 2: adjusted for age, sex, education level, residence, alcohol consumption, and income

*Abbreviations*: *CI* Confidence interval, *CVD* Cardiovascular disease, *HR* Hazard ratio, *NO*_*2*_ Nitrogen dioxide, *O*_*3*_ Ozone, *PM*_*1*_ Particulate matter with an aerodynamic diameter less than 1 μm, *PM*_*2.5*_ Particulate matter with an aerodynamic diameter less than 2.5 μm, *PM*_*10*_ Particulate matter with an aerodynamic diameter < 10 μm

### Interaction analysis of lifestyle and air pollution with incident CVD

**Fig. 3:**
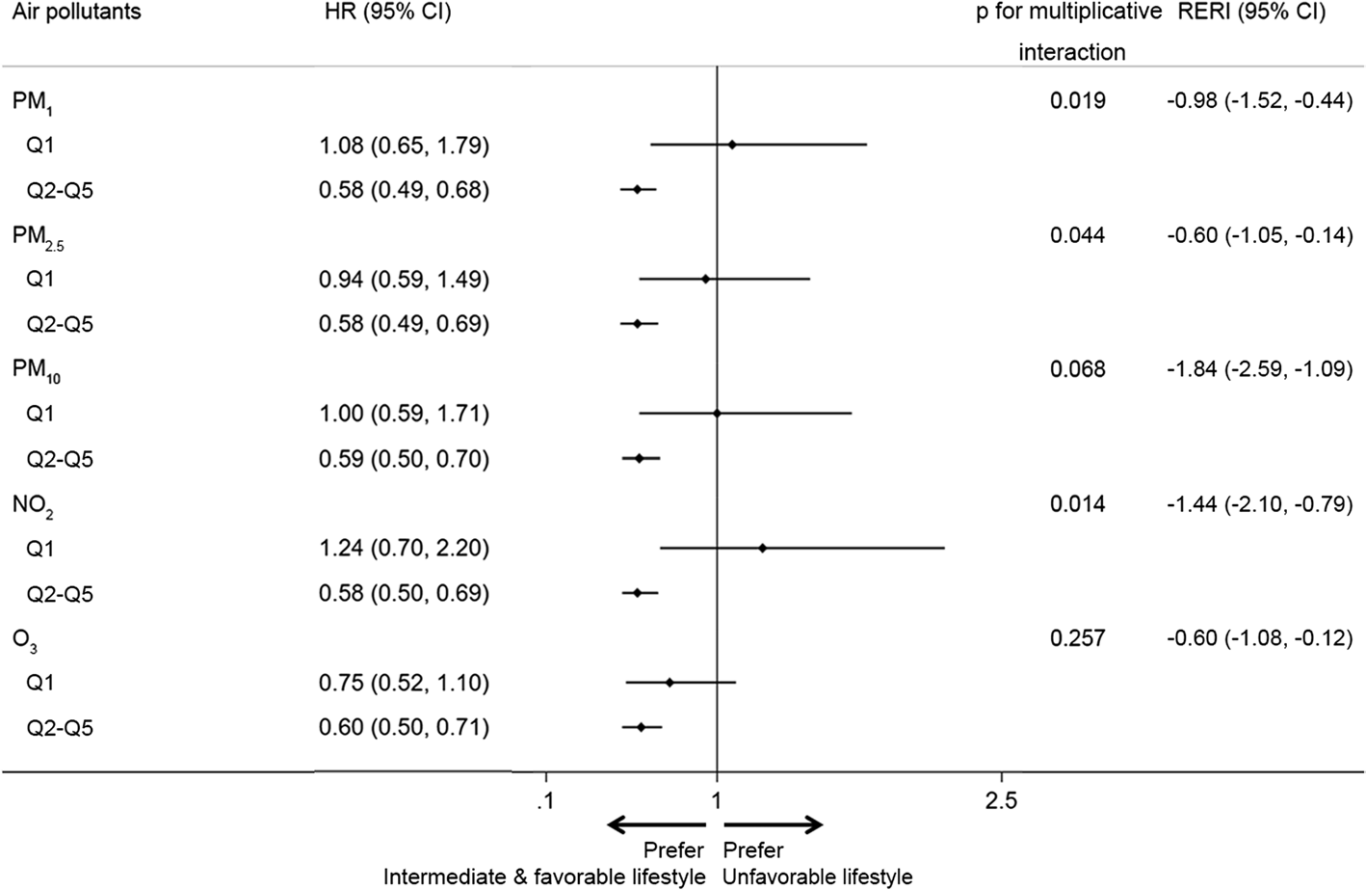
Multiplicative and additive interaction analysis of the effect of dichotomized lifestyle on the association between ambient air pollutant exposure and CVD. Model adjusted for age, sex, education, residence, alcohol consumption, and income. CI, confidence interval; CVD, cardiovascular disease; HR, hazard ratio; NO_2_, nitrogen dioxide; O_3_, ozone; PM_1_, particulate matter with an aerodynamic diameter less than 1 μm; PM_2.5_, particulate matter with an aerodynamic diameter less than 2.5 μm; PM_10_, particulate matter with an aerodynamic diameter < 10 μm; RERI, relative excess risk due to interaction

As shown in Fig. 3, multiplicative interactions were identified between different lifestyles and air pollutant exposure, mainly for PM_1_, PM_2.5_, and NO_2_ but not for PM_10_ and O_3_, on the risk of CVD (all *p* for multiplicative interaction: < 0.05) in analyses with dichotomous stratification for lifestyle. An additive interaction was identified between different lifestyles and air pollutant exposure on CVD risk, as the RERI (95% CI) was − 0.98 (95% CI: − 1.52 to − 0.44) for PM_1_, − 0.60 (95% CI: − 1.05 to − 0.14) for PM_2.5_, − 1.84 (95% CI: − 2.59 to − 1.09) for PM_10_, − 1.44 (95% CI: − 2.10 to − 0.79) for NO_2_, and − 0.60 (95% CI: − 1.08 to − 0.12) for O_3_. In subgroup analyses, the reduced risk of CVD from a healthy lifestyle at high levels (Q2–Q5) of air pollution exposure (mainly for PM) was significant in men (all *p* for interaction < 0.05, Additional file 1: Table S7). On the basis of Cox regression, when the means of all other variables in the sample were used, the incidence of CVD among adults with unfavorable lifestyle was higher than that among adults with intermediate and favorable lifestyle (Additional file 1: Fig. S6). Along with the increase in air pollutant concentration, the incidence of CVD among adults with intermediate and favorable lifestyles continually decreased compared to adults with unfavorable lifestyle (Additional file 1: Fig. S6).

### Sensitivity analyses

The results of the association between air pollutant exposure, lifestyle and risk of CVD were not substantially changed when we considered depressive symptoms and solid fuel use as covariates (Additional file 1: Tables S8-9). The replacement of diverse air pollutant exposure time frames or different definitions of lifestyle scores and categories also reached the same findings (Additional file 1: Tables S8-9). Moreover, the results were similar to the main findings in the subgroups of participants who had all covariates, were free of chronic lung disease, or did not change residence (Additional file 1: Tables S8-9). The findings of the interaction between lifestyle and exposure to air pollutants were stable across different time frames of air pollution exposure, various definitions of lifestyle scoring and categorization, and even when considering the competing risk of mortality (Additional file 1: Tables S10-14). The baseline information, exposure, and outcome between the included and excluded participants were similar (Additional file 1: Tables S15-16).

## Discussion

In this national cohort study in China, exposure to higher ambient air pollution (mainly for PM_1_, PM_2.5_, PM_10_, and NO_2_) was associated with a higher risk of CVD, with 7.4% to 14.3% of the association being mediated by lifestyle factors. Healthy lifestyles mitigated the adverse effect of exposure to high levels of air pollutant exposure on CVD, especially for exposure to PM_1_, PM_2.5_, NO_2_, and O_3_. Furthermore, the protective effect of a healthy lifestyle on CVD was stronger as the levels of air pollutant exposure increased. This national representative cohort study provides compelling evidence of getting closer to a healthy lifestyle to decrease the risk of air pollution on CVD. As the influence of air pollution in most countries will continue for some time to come, our findings provide some relief for middle-aged and elderly people who are exposed to air pollution and may have substantial implications for other countries that are developing appropriate responses to air pollution.

A systematic review reported that exposure to all major air pollutants except O_3_ was associated with an increased risk of myocardial infarction [29], similar to our findings. Modifiable risk factors have attracted much more attention in recent years and have gradually turned into a healthy lifestyle that is closely related to the individual. Many clinical trials or observational studies have explored individual-level ways to reduce the harmful cardiovascular effect caused by air pollution [10–12, 30–32], but the conclusions were limited. First, these studies estimating the impact of a single lifestyle factor on CVD often could not reflect the comprehensive lifestyle [10, 11, 30, 31]. Additionally, the mediating and interactive roles of a comprehensive lifestyle in the relationship between air pollutant exposure and CVD were also unclear. Our study employed a more comprehensive way of assessing lifestyle and a more quantitative approach to evaluating the role of lifestyle and its interaction with air pollution in incident CVD. To some extent, the results of the study could fill the gaps of previous studies and provide supporting evidence for addressing the cardiovascular harms of air pollution.

In our study, we found that the adverse effect of air pollutant exposure on CVD was alleviated when lifestyle factors were accounted for. In the mediation analysis, we found that 7.4% to 14.3% of the association between air pollutant exposure and CVD risk among Chinese adults could be explained by lifestyle factors. This effect was less than the previous mediating effect of hypertension on the effect of air pollution on CVD [9], probably because the comprehensive lifestyle assessment was more objective with a balance of each lifestyle factor than the individual assessment. The mediating role of lifestyle in the association between cardiovascular risk factors and CVD has been verified in many studies [33–35]. Our results further confirm that CVD events are attributed to air pollution mainly through deteriorating lifestyle factors, such as high blood pressure, elevated blood glucose, and blood lipids. It is also suggested that promoting healthy lifestyles alone is still insufficient to avoid the adverse effects of air pollution on CVD, and other protective measures are also needed. In the sensitivity analyses, we further adjusted for novel CVD risk factors, depressive status, and indoor air pollution, and the results were consistent. Attention to emotional health and indoor air pollution may also contribute to reducing the harmful impact of air pollution on CVD [36, 37]. In addition, we also considered nighttime sleep duration as one of the lifestyle factors, which further enriched the meaning of healthy lifestyles protecting against the negative effects of air pollutant exposure.

Another novel finding of our study is that significant interactions were found between air pollution exposure and healthy lifestyle regarding incident CVD, which was supported in previous studies [4, 32]. The results of marginal effect analyses indicated that the protective effect of a healthy lifestyle on the reduction of air pollution-related CVD was stronger as the healthy lifestyle score increased, and this effect was also more effective as the air pollution level increased. This is an interesting finding, since it provided the dose–response relationship between air pollution exposure and risk of CVD that can be protected by a healthy lifestyle. A similar study focused on the cardiovascular effect of physical activity as well as outdoor air pollution exposure and pointed out that physical activity may result in high CVD risk in participants with high levels of air pollution exposure compared to those exposed at low levels [10]. The reason for this inconsistency with the results of our study may be the monotony of lifestyle assessment. In our study, in addition to being more physically active, people with a healthy lifestyle also had an advantage in other cardiovascular health indicators, such as target blood pressure, blood glucose, and blood lipids. The results of the interaction between lifestyle and air pollution remain consistent across different air pollutant exposure time frames or across differently defined lifestyle scores and categories. Therefore, people with healthy lifestyle could also resist the adverse effects of air pollution on CVD even under high exposure to air pollution.

Several studies have focused on the impacts of air pollutant inhalation and deposition on the cardiovascular system [38–40]. In a direct manner, air pollutants can pass directly through the alveoli into the bloodstream and eventually deposit on blood vessel walls, leading to endothelial dysfunction, vasoconstriction, and thrombosis [38, 39]. In an indirect way, air pollutants may induce oxidative stress and systemic inflammatory responses, leading to autonomic dysfunction and exacerbating the progression of CVD [40]. The cardiovascular benefits of adhering to a healthy lifestyle are due to its ability to reduce cardiovascular risk factors across multiple dimensions. There is evidence that regular physical activity can increase tolerance to the adverse effects of air pollution exposure [41]. Moreover, overall lifestyle improvement can achieve cardiovascular benefits by reducing cardiovascular metabolic risks [32] and mitigating systemic inflammation, thereby resisting the harm caused by air pollution [42].

Our results support public health efforts aimed at reducing the burden of CVD. A healthy lifestyle can effectively reduce the risk of CVD, even among those exposed to high levels of air pollution. This has significant strategic implications for the prevention and control of CVD in countries with high exposure to air pollution, in addition to public health policies that encourage the reduction of air pollutant intake by reducing air pollutant emissions and increasing air filtration systems, on the other hand, and, more importantly, focus on lifestyle improvements at the individual level.

Our study has several advantages. First, we used a cohort with a median follow-up of 7 years to clarify the mediating role of a comprehensive healthy lifestyle in the relationship between exposure to various air pollutants and CVD risk. We also identified the interaction between a healthy lifestyle and exposure to different air pollutants. Additionally, we conducted a series of sensitivity analyses, including different air pollution exposure time frames and classification methods for lifestyle factors, to strengthen our conclusions. There are several limitations to our study. First, given the protection policies of the CHARLS for sensitive information such as an individual’s residential address, we can only estimate exposure based on the district in which the participant lives, which may lead to misclassification of exposure. Nevertheless, we employed the accurate measurement of air pollution as much as possible and introduced indoor air pollution as a covariate in the sensitivity analysis to supplement this deficiency. Second, the diagnosis of CVD was based on self-reported data derived from the physician diagnosed, which may also have some degree of bias. However, high consistency between self-reported coronary artery disease and medical records was confirmed by the English Longitudinal Study of Ageing researchers [43]. Third, the assessment of lifestyle in this study was based on baseline levels rather than being dynamic and therefore may not represent the long-term status. However, the lifestyles of middle-aged and older adults tend to be more fixed, with less likelihood of significant changes, thus causing minimal fluctuation in the groupings. Finally, previous studies have shown that nutrient intake also affects the relationship between air pollution exposure and CVD [30, 31], but due to the lack of dietary data in the CHARLS, the effect of dietary factors could not be estimated in this study. Nevertheless, differences in diet can also be partially reflected in lifestyle factors, such as lipid profiles, blood glucose, blood pressure, and BMI. Future studies are needed to further explore the impact of diet on the relationship between air pollution exposure and CVD.

## Conclusions

Based on a national cohort in China, we found that the association between ambient air pollutant exposure and CVD was partially mediated by lifestyle. Adherence to a healthy lifestyle can significantly reduce the incidence of CVD, especially for people exposed to high levels of air pollution. This study highlights the importance of lifestyle improvement in reducing the burden of CVD disease, providing an effective way to mitigate the impact of air pollution. Proactive policies are needed to address the health problems caused by air pollution, and in addition to tackling the source of air pollution, individual-level protective measures to maintain a healthy lifestyle are also needed.

### Supplementary Information

**Additional file 1: Method S1.** Ambient air pollution exposure acquisition. **Figure S1.** Sampling procedure. **Figure S2.** Study flowchart. **Figure S3.** The association of different lifestyle factors. **Figure S4.** (a) The proportion of single ideal factor in different lifestyle groups. (b) The proportion of ideal factors in different lifestyle groups. **Figure S5.** Directed acyclic graph. **Figure S6.** The marginal effect of lifestyle on CVD and in the relationship between ambient air pollutant exposure and CVD. **Table S1.** The score criteria of different lifestyle factors. **Table S2.** The exposure level of different air pollutants among the study population. **Table S3.** The exposure level by quintile of air pollutant. **Table S4.** The HRs (95% CIs) of the associations between lifestyle and CVD with and without adjustment for ambient air pollutant exposure. **Table S5.** Joint effects of lifestyle and air pollutant exposure on the incidence of CVD. **Table S6.** The HRs (95% CIs) of incident CVD associated with each lifestyle factor at different levels of air pollutant exposure. **Table S7.** Subgroup analysis of the additive interactions analysis of the effect of dichotomized lifestyle on the association between ambient air pollutant exposure and CVD in high air pollutant exposure levels (Q2–Q5). **Table S8.** The HRs (95% CIs) of associations between air pollutant exposure (per 10 μg/m^3^ increase) and incident CVD, and the mediation effect of lifestyle categories on air pollution and CVD in different sensitivity analysis models. **Table S9.** The HRs (95% CIs) of the association between ambient air pollutant exposure (per 10 μg/m^3^ increase) and CVD in different lifestyle categories in different sensitivity analysis models. **Table S10.** Multiplicative and additive interaction analysis of the effect of dichotomized lifestyle on the association between time-varying ambient air pollutant exposure and CVD. **Table S11.** Multiplicative and additive interaction analysis of the effect of dichotomized lifestyle on the association between 3 years of ambient air pollutant exposure and CVD. **Table S12.** Multiplicative and additive interaction analysis of the effect of dichotomized lifestyle considering new categories and nighttime sleep duration on the association between ambient air pollutant exposure and CVD. **Table S13.** Multiplicative and additive interaction analysis of the effect of dichotomized lifestyle considering new assignment of lifestyle categories on the association between ambient air pollutant exposure and CVD. **Table S14.** The subdistribution HRs (sHRs, 95% CI) of the associations between ambient air pollutant exposure (per 10 μg/m^3^) and CVD in different lifestyle categories. **Table S15.** Baseline characteristics of included and excluded participants. **Table S16.** Baseline characteristics of included participants and those without lifestyle scores.

## Data Availability

The data can be accessed from the China Health and Retirement Longitudinal Study (CHARLS) (http://charls.pku.edu.cn/) with application.

## Abbreviations

BMI: Body mass index
CES-D: Center for epidemiologic studies-depression
CHARLS: China health and retirement longitudinal study
CI: Confidence interval
CVD: Cardiovascular disease
DAG: Directed acyclic graph
HR: Hazard ratio
NO_2_: Nitrogen dioxide
O_3_: Ozone
PM_1_: Particles with diameters ≤ 1.0 μm
PM_10_: Particles with diameters ≤ 10 μm
PM_2.5_: Particles with diameters ≤ 2.5 μm
RERI: Relative excess risk due to interactions
