# Supplementary material for: The role of lifestyle in the association between long-term ambient air pollution exposure and cardiovascular disease: a national cohort study in China
Source: BMC Med. 2024 Mar 5;22:93. doi: 10.1186/s12916-024-03316-z (PMC10913402; doi:10.1186/s12916-024-03316-z)
Supplement: Supplementary file 1 — Additional file 1: Method S1. Ambient air pollution exposure acquisition. Figure S1. Sampling procedure. Figure S2. Study flowchart. Figure S3. The association of different lifestyle factors. Figure S4. (a) The proportion of single ideal factor in different lifestyle groups. (b) The proportion of ideal factors in different lifestyle groups. Figure S5. Directed acyclic graph. Figure S6. The marginal effect of lifestyle on CVD and in the relationship between ambient air pollutant exposure and CVD. Table S1. The score criteria of different lifestyle factors. Table S2. The exposure level of different air pollutants among the study population. Table S3. The exposure level by quintile of air pollutant. Table S4. The HRs (95% CIs) of the associations between lifestyle and CVD with and without adjustment for ambient air pollutant exposure. Table S5. Joint effects of lifestyle and air pollutant exposure on the incidence of CVD. Table S6. The HRs (95% CIs) of incident CVD associated with each lifestyle factor at different levels of air pollutant exposure. Table S7. Subgroup analysis of the additive interactions analysis of the effect of dichotomized lifestyle on the association between ambient air pollutant exposure and CVD in high air pollutant exposure levels (Q2–Q5). Table S8. The HRs (95% CIs) of associations between air pollutant exposure (per 10 μg/m3 increase) and incident CVD, and the mediation effect of lifestyle categories on air pollution and CVD in different sensitivity analysis models. Table S9. The HRs (95% CIs) of the association between ambient air pollutant exposure (per 10 μg/m3 increase) and CVD in different lifestyle categories in different sensitivity analysis models. Table S10. Multiplicative and additive interaction analysis of the effect of dichotomized lifestyle on the association between time-varying ambient air pollutant exposure and CVD. Table S11. Multiplicative and additive interaction analysis of the effect of dichotomized lifestyle on the assoc [file 12916_2024_3316_MOESM1_ESM.docx]

**The role of lifestyle in the association between long-term ambient air pollution exposure and cardiovascular disease: a national cohort study in China**

**Method S1.** Ambient air pollution exposure acquisition

**Fig. S1.** Sampling procedure

**Fig. S2.** Study flowchart

**Fig. S3.** The association of different lifestyle factors

**Fig. S4.** (a) The proportion of single ideal factor in different lifestyle groups. (b) The proportion of ideal factors in different lifestyle groups

**Fig. S5.** Directed acyclic graph

**Fig. S6.** The marginal effect of lifestyle on CVD and in the relationship between ambient air pollutant exposure and CVD

**Table S1.** The score criteria of different lifestyle factors

**Table S2.** The exposure level of different air pollutants among the study population

**Table S3.** The exposure level by quintile of air pollutant

**Table S4.** The HRs (95% CIs) of the associations between lifestyle and CVD with and without adjustment for ambient air pollutant exposure

**Table S5.** Joint effects of lifestyle and air pollutant exposure on the incidence of CVD

**Table S6.** The HRs (95% CIs) of incident CVD associated with each lifestyle factor at different levels of air pollutant exposure

**Table S7.** Subgroup analysis of the additive interactions analysis of the effect of dichotomized lifestyle on the association between ambient air pollutant exposure and CVD in high air pollutant exposure levels (Q2–Q5)

**Table S8.** The HRs (95% CIs) of associations between air pollutant exposure (per 10 μg/m^3^ increase) and incident CVD, and the mediation effect of lifestyle categories on air pollution and CVD in different sensitivity analysis models

**Table S9.** The HRs (95% CIs) of the association between ambient air pollutant exposure (per 10 μg/m^3^ increase) and CVD in different lifestyle categories in different sensitivity analysis models

**Table S10.** Multiplicative and additive interaction analysis of the effect of dichotomized lifestyle on the association between time-varying ambient air pollutant exposure and CVD

**Table S11.** Multiplicative and additive interaction analysis of the effect of dichotomized lifestyle on the association between three years of ambient air pollutant exposure and CVD

**Table S12.** Multiplicative and additive interaction analysis of the effect of dichotomized lifestyle considering new categories and nighttime sleep duration on the association between ambient air pollutant exposure and CVD

**Table S13.** Multiplicative and additive interaction analysis of the effect of dichotomized lifestyle considering new assignment of lifestyle categories on the association between ambient air pollutant exposure and CVD

**Table S14.** The subdistribution HRs (sHRs, 95% CI) of the associations between ambient air pollutant exposure (per 10 μg/m^3^) and CVD in different lifestyle categories

**Table S15.** Baseline characteristics of included and excluded participants

**Table S16.** Baseline characteristics of included participants and those without lifestyle scores

**Method S1.** Ambient air pollution exposure acquisition

Daily concentrations of PM_1_ and PM_2.5_ were estimated at a high spatial resolution (0.1°× 0.1°) by satellite-based spatio-temporal models. We used daily satellite-based aerosol optical depths (AOD) data derived from two types of Moderate Resolution Imaging Spectroradiometer algorithms, Dark Target and Deep Blue.^1, 2^ Then, we predicted daily PM_1_ and PM_2.5_ concentrations for the participants’ communities with the combination of ground monitoring, meteorology, land use, and other spatial predictors using random forest models. The 10-fold cross-validation tests showed that the adjusted R^2^ value (root mean squared error) was 71% (13.0 µg/m^3^) for monthly PM_1_ prediction and 75% (15.08 µg/m^3^) for monthly PM_2.5_ prediction. The concentrations of PM_10_, NO_2_, and O_3_ were derived from appropriately located air monitors in the district where each study participant lived.^3-5^ The air monitoring stations were placed far away from major traffic roads, gas stations, industrial fuel exhaust outlets, and landfills to ensure that the measurements collected were more likely to reflect background air pollution levels. The monitoring strictly followed the procedures set by the State Environmental Protection Administration of China [State Environmental Protection Administration of China. Standardized environmental monitoring and analysis methods. Beijing: State Environmental Protection Administration, 1992.]. We calculated daily concentrations of PM_10_ using β-attenuation, NO_2_ using chemiluminescence, and O_3_ using ultraviolet photometry. Daily concentrations of PM_10_ and NO_2_ and max 8-h average O_3_ concentrations were estimated using hour-long measurements throughout a day with at least 75.0% of 1–h values available. After that, data from inversion data of ambient air pollutants were used to draw the map of air pollutants across China.^6-10^

**References:**

1 Chen G, Knibbs LD, Zhang W, et al. Estimating spatiotemporal distribution of PM1 concentrations in China with satellite remote sensing, meteorology, and land use information. *Environ Pollut* 2018;233:1086-1094.

2 Chen G, Li S, Knibbs LD, et al. A machine learning method to estimate PM2.5 concentrations across China with remote sensing, meteorological and land use information. *Sci Total Environ* 2018;636:52-60.

3 Zhan Y, Luo Y, Deng X, et al. Satellite-Based Estimates of Daily NO2 Exposure in China Using Hybrid Random Forest and Spatiotemporal Kriging Model. *Environ Sci Technol* 2018;52:4180-4189.

4 Chen G, Wang Y, Li S, et al. Spatiotemporal patterns of PM10 concentrations over China during 2005-2016: A satellite-based estimation using the random forests approach. *Environ Pollut* 2018;242:605-613.

5 Chen G, Chen J, Dong G, Yang B, Liu Y, Lu T, et al. Improving satellitebased estimation of surface ozone across China during 2008–2019 using iterative random forest model and high-resolution grid meteorological data. *Sustain. Cities Soc* 2021;69:102807

6 Wei J, Li Z, Guo J, et al. Satellite-Derived 1-km-Resolution PM1 Concentrations from 2014 to 2018 across China. *Environ Sci Technol* 2019;53:13265-13274.

7 Wei J, Li Z, Lyapustin A, Sun L, Peng Y, Xue W, et al. Reconstructing 1-km-resolution high-quality PM2.5 data records from 2000 to 2018 in China: spatiotemporal variations and policy implications. *Remote Sensing of Environment* 2021;252:112136.

8 Wei J, Li Z, Xue W, et al. The ChinaHighPM10 dataset: generation, validation, and spatiotemporal variations from 2015 to 2019 across China. *Environ Int* 2021;146:106290.

9 Wei J, Li Z, Li K, Dickerson R, Pinker R, Wang J, et al. Full-coverage mapping and spatiotemporal variations of ground-level ozone (O3) pollution from 2013 to 2020 across China. *Remote Sensing of Environment* 2022;270, 112775.

10 Wei J, Li Z, Wang J, Li C, Gupta P, Cribb M. Ground-level gaseous pollutants (NO2, SO2, and CO) in China: daily seamless mapping and spatiotemporal variations. *Atmospheric Chemistry and Physics* 2023;23:1511–1532.


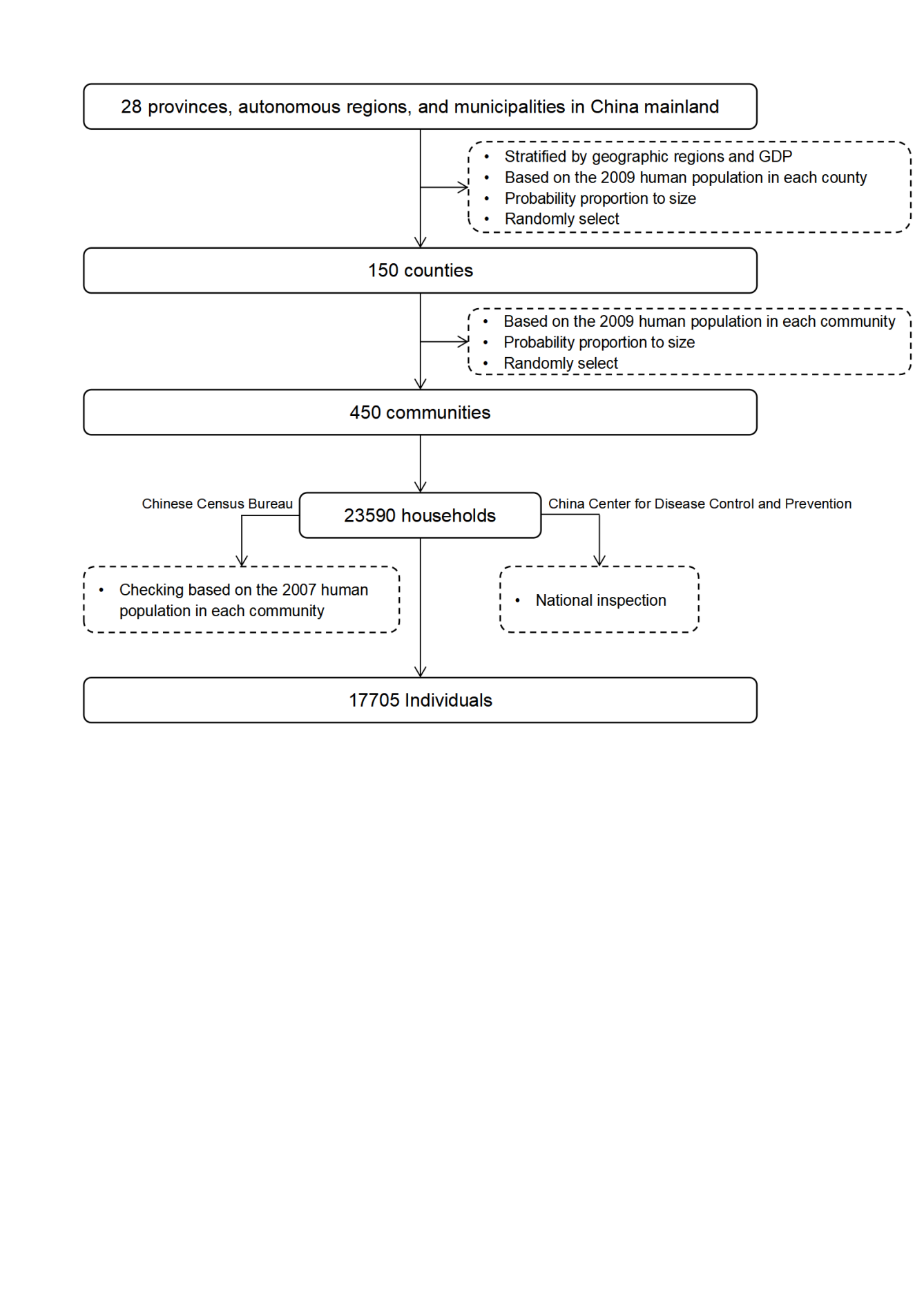


**Fig. S1.** Sampling procedure


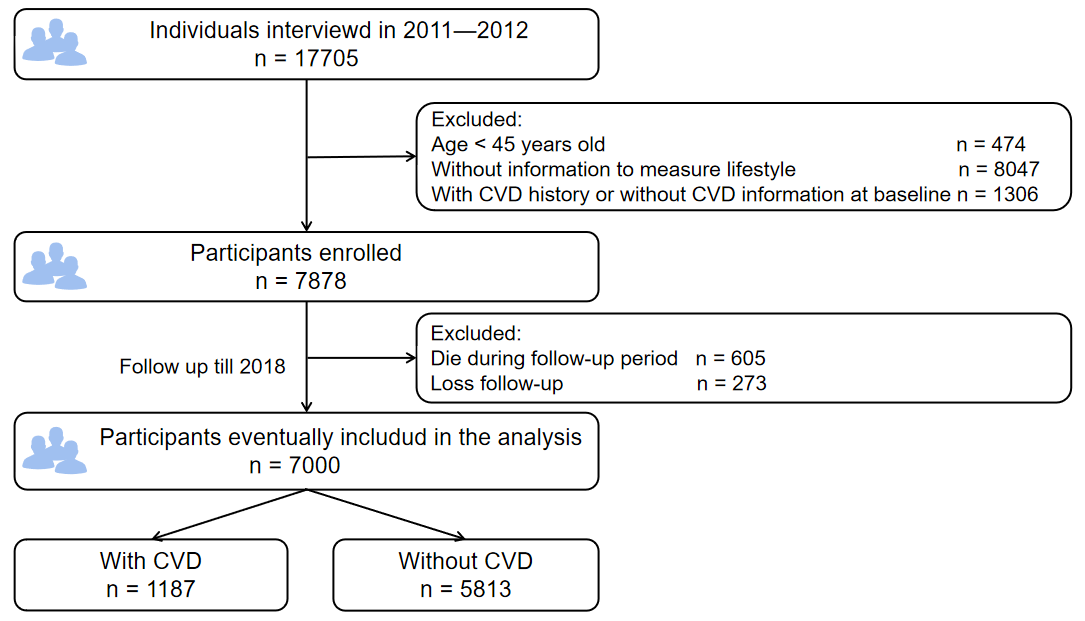


**Fig. S2.** Study flowchart

CVD cardiovascular disease.


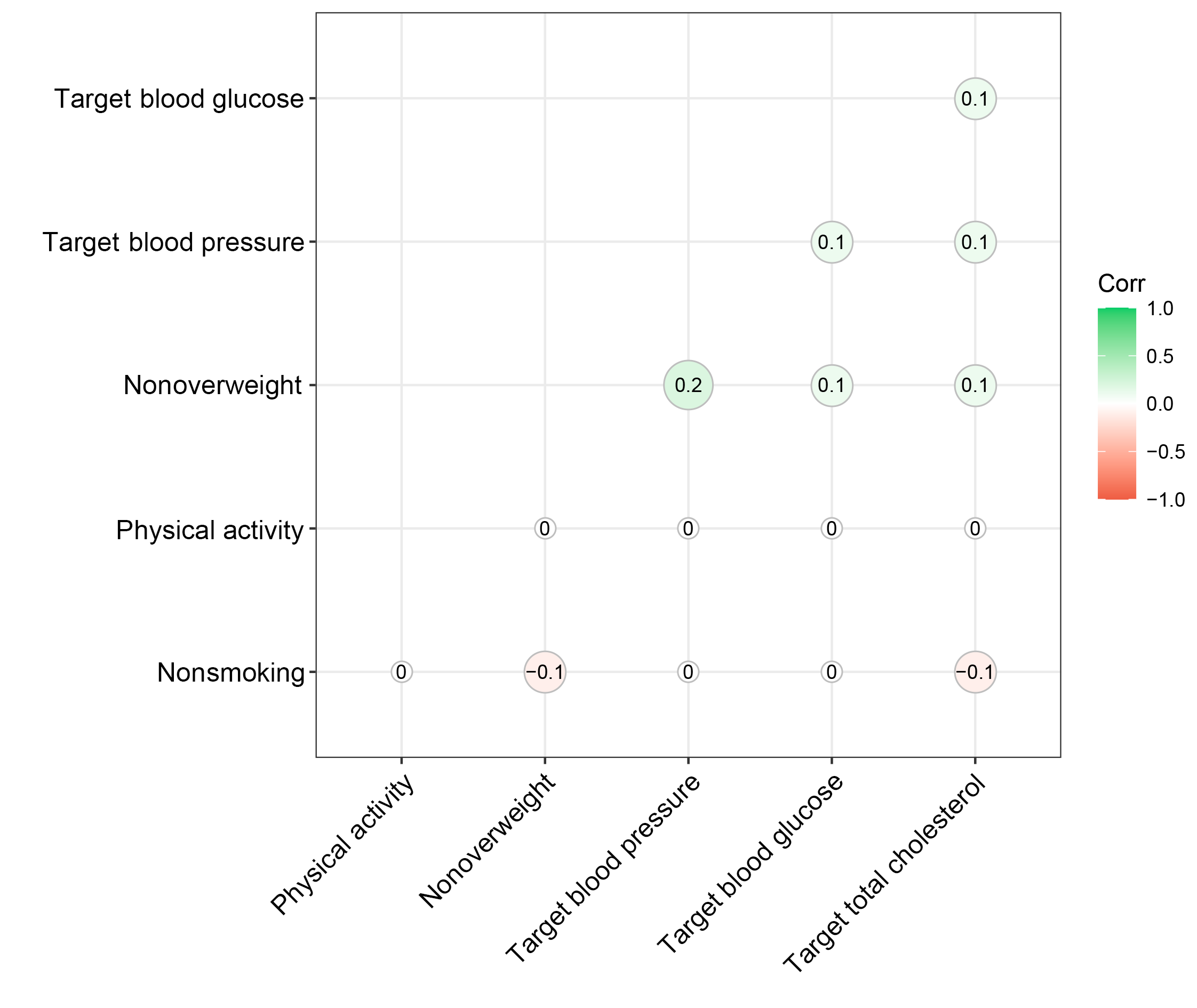


**Fig. S3.** The association of different lifestyle factors


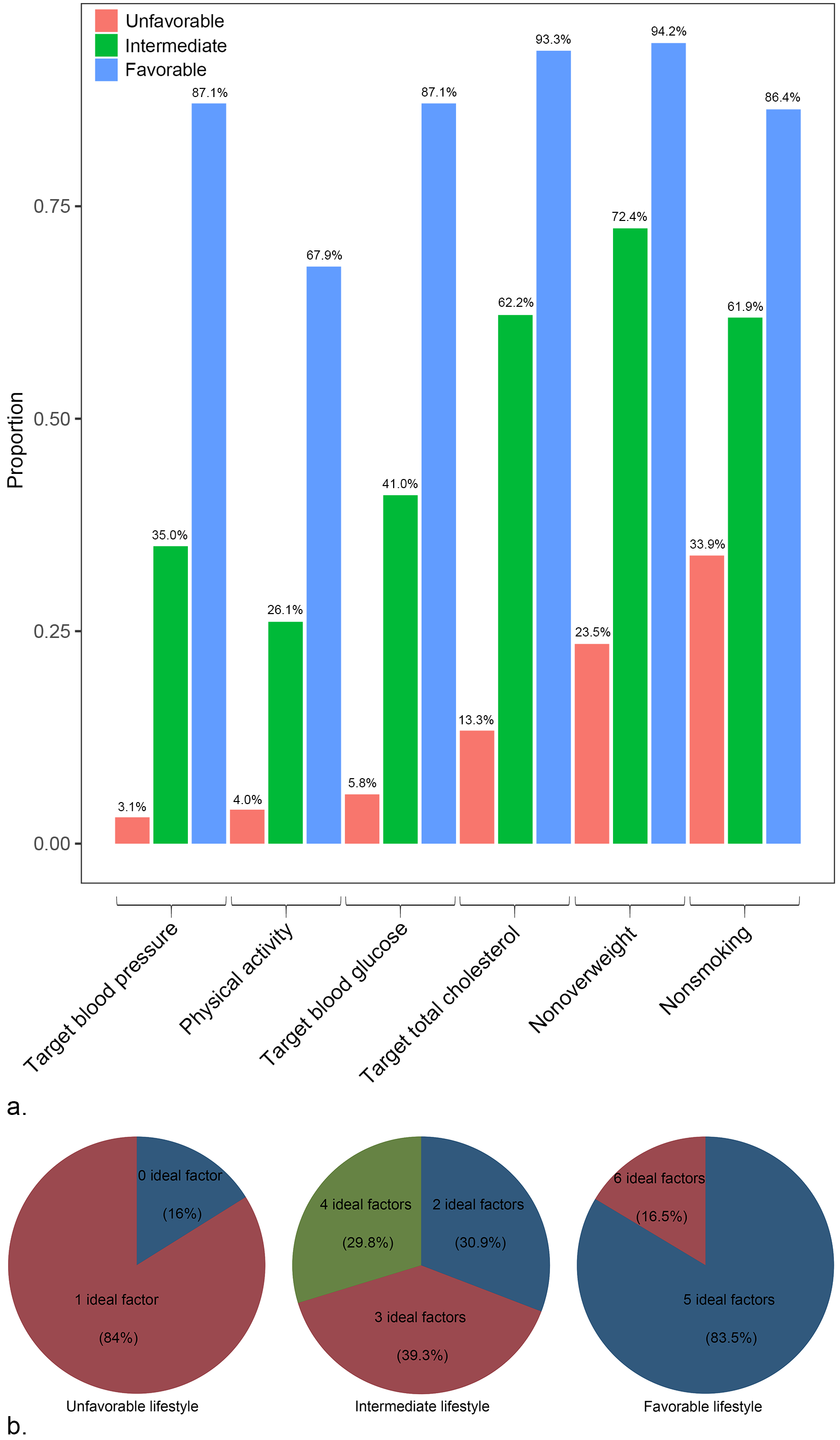


**Fig. S4.** (a) The proportion of single ideal factor in different lifestyle groups. (b) The proportion of ideal factors in different lifestyle groups


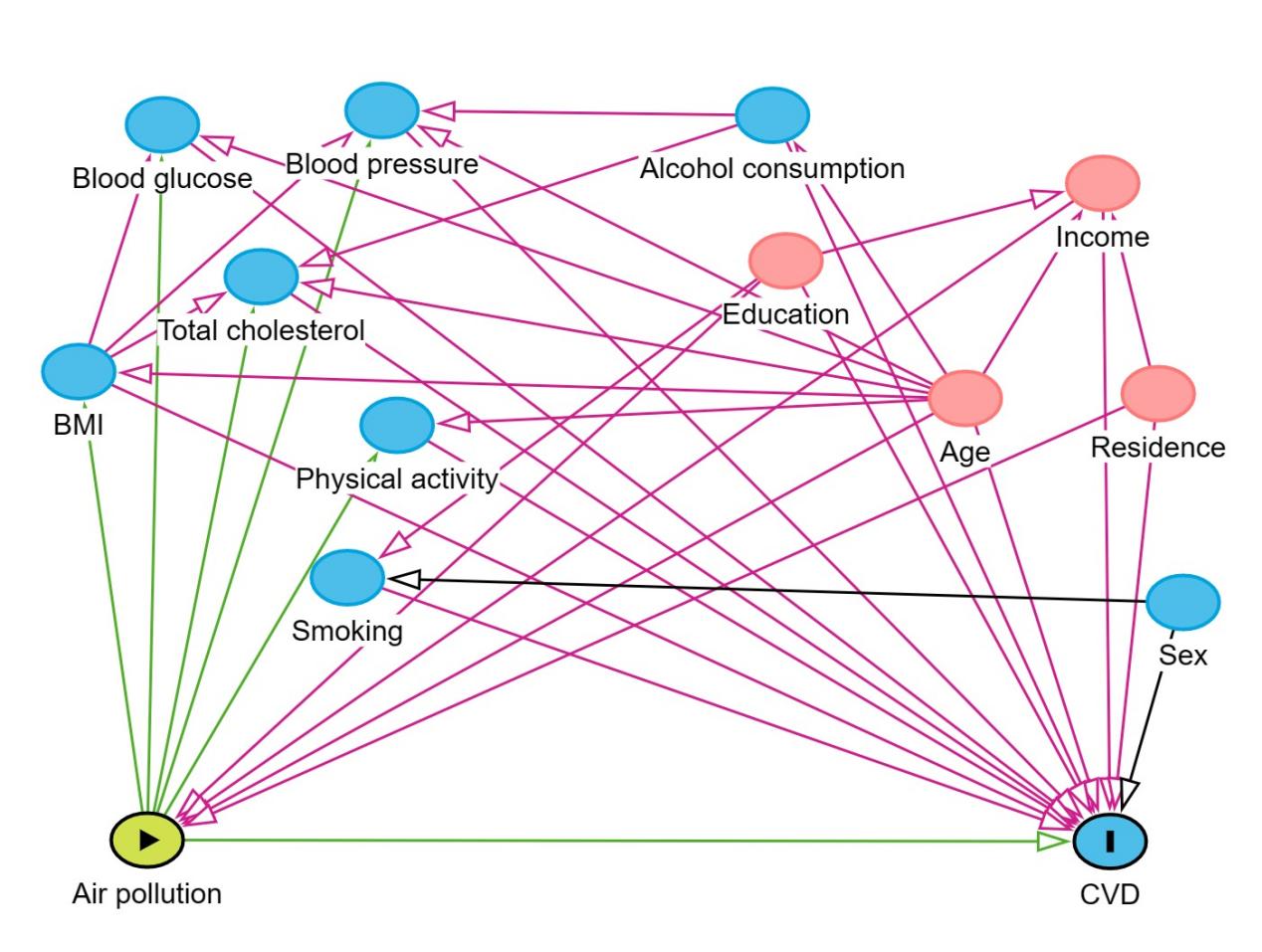


**Fig. S5.** Directed acyclic graph

BMI body-mass index, CVD cardiovascular disease.


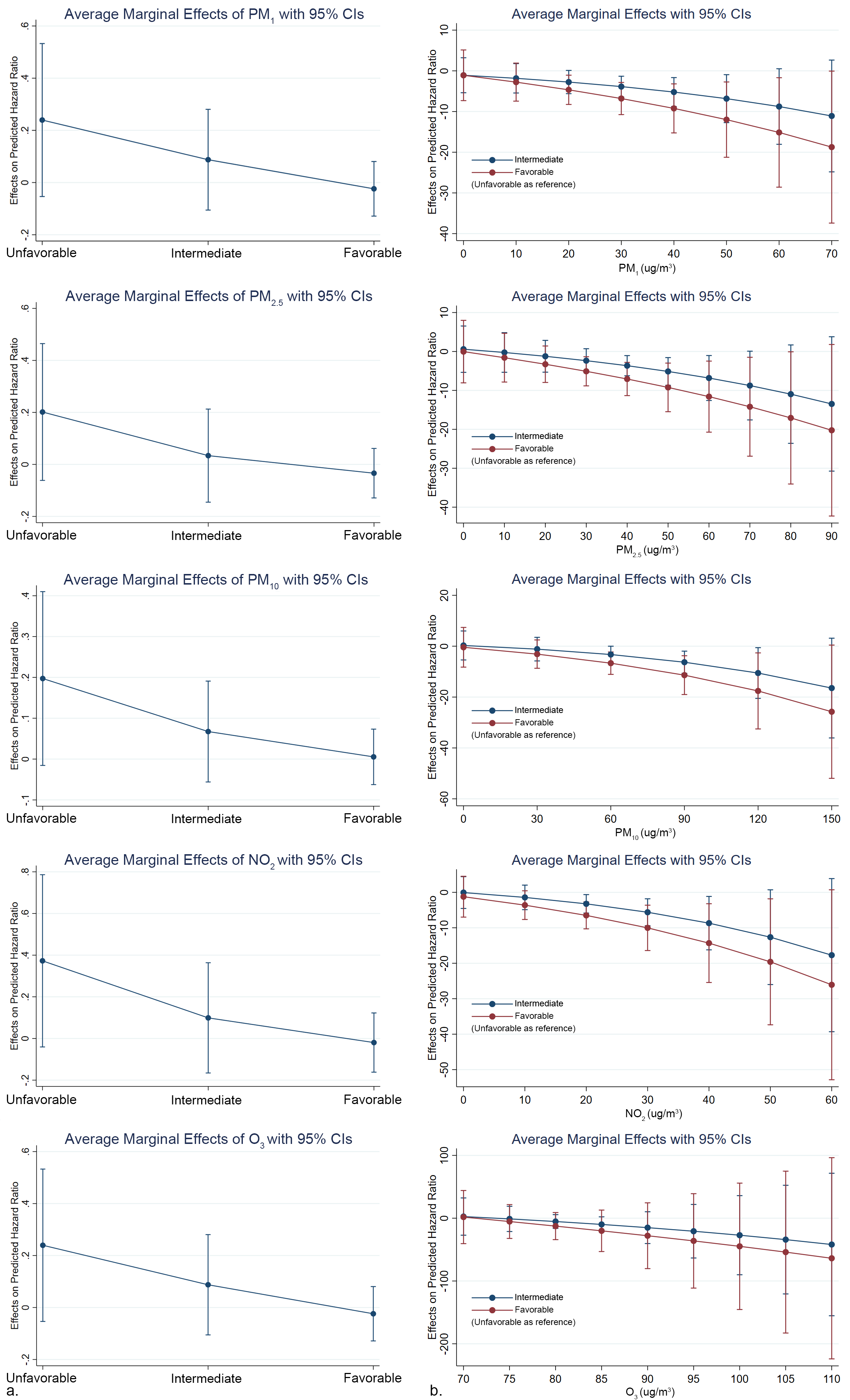


**Fig. S6.** The marginal effect of lifestyle on CVD and in the relationship between ambient air pollutant exposure and CVD

(a) The marginal effect of lifestyle on CVD. (b) The marginal effect of lifestyle on the association between ambient air pollutant exposure and CVD.

CI confidence interval, CVD cardiovascular disease, HR hazard ratio, NO_2_ nitrogen dioxide, O_3_ ozone, PM_1_ particulate matter with an aerodynamic diameter less than 1 μm, PM_2.5_ particulate matter with an aerodynamic diameter less than 2.5 μm, PM_10_ particulate matter with an aerodynamic diameter <10 μm.

**Table S1.** The score criteria of different lifestyle factors

|  | Score | | | | |
| --- | --- | --- | --- | --- | --- |
|  | Two categories [1] | | Three categories^a^ [2] | | |
|  | 0 | 1 | 0 | 1 | 2 |
| Blood pressure, mmHg | SBP≥120 or DBP≥80 | SBP<120 and DBP<80 | SBP ≥ 140 or DBP ≥ 90 | SBP 120–139 or  DBP 80–89 | SBP < 120 and DBP < 80 |
| Blood glucose, mg/dL | ≥ 100 | < 100 | ≥ 126 | 100–125 | < 100 |
| TC, mg/dL | ≥ 200 | < 200 | ≥ 240 | 200–239 | < 200 |
| BMI, kg/m^2^ | ≥ 25 | < 25 | ≥ 30 | 25–29.9 | < 25 |
| Smoking | Yes | None | Yes | Former ≤ 12 month | Never or quit > 12 month |
| Physical activity [3] | None | 30 minutes of vigorous exercise (including heavy lifting, digging, ploughing, aerobics, fast bicycling, and cycling with a heavy load) or moderate physical activity (including carrying light loads, bicycling at a regular pace, or mopping the floor) at least three times a week | None | 1–29 minutes of vigorous exercise (including heavy lifting, digging, ploughing, aerobics, fast bicycling, and cycling with a heavy load) or moderate physical activity (including carrying light loads, bicycling at a regular pace, or mopping the floor) at least one time a week | 30 minutes of vigorous exercise (including heavy lifting, digging, ploughing, aerobics, fast bicycling, and cycling with a heavy load) or moderate physical activity (including carrying light loads, bicycling at a regular pace, or mopping the floor) at least three times a week |
| Nighttime sleep duration, hours^a^ | <6 or >8 | 6–8 | - | - | - |

^a^For sensitivity analysis

BMI body-mass index, DBP diastolic blood pressure, SBP systolic blood pressure, TC total cholesterol.

**References:**

1. Chen W, Wang X, Chen J, et al. Household air pollution, adherence to a healthy lifestyle, and risk of cardiometabolic multimorbidity: Results from the China health and retirement longitudinal study. *Sci Total Environ* 2023;855:158896.
2. Xia X, Qiu C, Rizzuto D, Grande G, Laukka EJ, Fratiglioni L, Guo J, Vetrano DL. The age-dependent association of Life's Simple 7 with transitions across cognitive states after age 60. *J Intern Med* 2023;294(2):191-202.
3. National health commission of the People’ s Republic of China. *Healthy China Initiative (2019-2030)* 2019. http://www.gov.cn/xinwen/2019-07/15/content_5409694.htm (In Chinese, 10 Aug 2023)

**Table S2.** The exposure level of different air pollutants among the study population

|  | Minimum | 10% | 25% | 50% | 75% | 90% | Maximum | Mean (SD) | Median (IQR) |
| --- | --- | --- | --- | --- | --- | --- | --- | --- | --- |
| Total (N = 7000) | | | | | | | | | |
| PM_1_, μg/m^3^ | 13.61 | 20.44 | 29.26 | 41.12 | 52.45 | 57.75 | 68.84 | 39.97 (13.80) | 41.12 (23.19) |
| PM_2.5_, μg/m^3^ | 17.54 | 30.45 | 41.03 | 52.95 | 65.73 | 72.70 | 80.97 | 52.61 (15.85) | 52.95 (24.7) |
| PM_10_, μg/m^3^ | 34.52 | 50.29 | 73.52 | 93.12 | 112.40 | 134.16 | 149.58 | 93.44 (28.05) | 93.12 (38.88) |
| NO_2_, μg/m^3^ | 9.30 | 15.68 | 20.46 | 26.99 | 37.71 | 44.51 | 57.27 | 29.22 (10.79) | 26.99 (17.25) |
| O_3_, μg/m^3^ | 78.50 | 87.25 | 89.55 | 96.37 | 100.60 | 103.71 | 107.03 | 95.32 (6.48) | 96.37 (11.05) |
| Unfavorable lifestyle (N = 818) | | | | | | | | | |
| PM_1_, μg/m^3^ | 13.61 | 22.35 | 31.04 | 44.08 | 53.74 | 59.16 | 68.84 | 42.01 (13.63) | 44.08 (22.7) |
| PM_2.5_, μg/m^3^ | 17.54 | 33.55 | 43.86 | 54.37 | 66.62 | 72.90 | 80.87 | 54.57 (15.61) | 54.37 (22.76) |
| PM_10_, μg/m^3^ | 34.52 | 59.40 | 78.74 | 94.91 | 117.94 | 135.98 | 148.74 | 97.48 (27.98) | 94.91 (39.2) |
| NO_2_, μg/m^3^ | 10.16 | 18.08 | 22.00 | 29.28 | 40.64 | 46.12 | 57.27 | 31.15 (10.74) | 29.28 (18.64) |
| O_3_, μg/m^3^ | 78.50 | 87.24 | 90.21 | 97.16 | 101.24 | 104.32 | 107.03 | 95.77 (7.05) | 97.16 (11.03) |
| Intermediate lifestyle (N = 5308) | | | | | | | | | |
| PM_1_, μg/m^3^ | 13.61 | 20.15 | 29.14 | 41.01 | 52.45 | 57.33 | 68.84 | 39.87 (13.82) | 41.01 (23.31) |
| PM_2.5_, μg/m^3^ | 17.54 | 30.45 | 41.16 | 52.95 | 65.73 | 72.70 | 80.97 | 52.61 (15.82) | 52.95 (24.57) |
| PM_10_, μg/m^3^ | 34.52 | 50.20 | 73.54 | 93.12 | 112.40 | 134.16 | 149.58 | 93.32 (27.99) | 93.12 (38.86) |
| NO_2_, μg/m^3^ | 9.30 | 15.68 | 20.46 | 26.99 | 37.68 | 44.51 | 57.27 | 29.20 (10.76) | 26.99 (17.22) |
| O_3_, μg/m^3^ | 78.50 | 87.25 | 89.55 | 96.27 | 100.57 | 103.71 | 107.03 | 95.26 (6.46) | 96.27 (11.02) |
| Favorable lifestyle (N = 874) | | | | | | | | | |
| PM_1_, μg/m^3^ | 13.61 | 20.15 | 28.38 | 36.74 | 50.62 | 56.52 | 68.06 | 38.69 (13.64) | 36.74 (22.24) |
| PM_2.5_, μg/m^3^ | 17.54 | 29.11 | 38.36 | 50.41 | 63.74 | 72.66 | 80.87 | 50.83 (16.08) | 50.41 (25.38) |
| PM_10_, μg/m^3^ | 34.52 | 50.15 | 69.65 | 87.58 | 108.56 | 134.16 | 148.74 | 90.33 (28.05) | 87.58 (38.91) |
| NO_2_, μg/m^3^ | 9.30 | 14.97 | 19.32 | 25.13 | 37.65 | 43.21 | 57.27 | 27.57 (10.74) | 25.13 (18.33) |
| O_3_, μg/m^3^ | 78.50 | 87.26 | 89.55 | 96.21 | 100.16 | 103.38 | 107.03 | 95.30 (6.11) | 96.21 (10.61) |

IQR interquartile range, NO_2_ nitrogen dioxide, O_3_ ozone, PM_1_ particulate matter with an aerodynamic diameter less than 1 μm, PM_2.5_ particulate matter with an aerodynamic diameter less than 2.5 μm, PM_10_ particulate matter with an aerodynamic diameter <10 μm, SD standard deviation.

**Table S3.** The exposure level by quintile of air pollutant

|  | Minimum | Maximum | Mean (SD) | Median (IQR) |
| --- | --- | --- | --- | --- |
| PM_1_, μg/m^3^ |  |  |  |  |
| Q1 (n=1384) | 13.61 | 25.70 | 20.25 (3.92) | 20.44 (7.24) |
| Q2–Q5 (n=5616) | 26.06 | 68.84 | 44.83 (10.69) | 44.93 (18.63) |
| PM_2.5_, μg/m^3^ |  |  |  |  |
| Q1 (n=1406) | 17.54 | 38.15 | 29.31 (6.04) | 30.45 (10.68) |
| Q2–Q5 (n=5594) | 38.21 | 80.97 | 58.47 (11.60) | 58.28 (21.29) |
| PM_10_, μg/m^3^ |  |  |  |  |
| Q1 (n=1412) | 34.52 | 66.89 | 53.81 (9.37) | 50.29 (14.41) |
| Q2–Q5 (n=5588) | 67.39 | 149.58 | 103.45 (21.59) | 101.18 (32.46) |
| NO_2_, μg/m^3^ |  |  |  |  |
| Q1 (n=1401) | 9.30 | 19.15 | 15.72 (2.48) | 15.68 (3.60) |
| Q2–Q5 (n=5599) | 19.31 | 57.27 | 32.60 (9.32) | 30.58 (16.54) |
| O_3_, μg/m^3^ |  |  |  |  |
| Q1 (n=1370) | 78.50 | 88.70 | 86.14 (2.96) | 87.25 (2.47) |
| Q2–Q5 (n=5630) | 88.84 | 107.03 | 97.56 (4.96) | 98.08 (7.66) |

IQR interquartile range, NO_2_ nitrogen dioxide, O_3_ ozone, PM_1_ particulate matter with an aerodynamic diameter less than 1 μm, PM_2.5_ particulate matter with an aerodynamic diameter less than 2.5 μm, PM_10_ particulate matter with an aerodynamic diameter <10 μm, SD standard deviation.

**Table S4.** The HRs (95% CIs) of the associations between lifestyle and CVD with and without adjustment for ambient air pollutant exposure

|  | Unadjusted for air pollution | Adjusted for air pollution* |
| --- | --- | --- |
| Lifestyle |  |  |
| Unfavorable | Reference | Reference |
| Intermediate | 0.65 (0.56–0.76) | 0.68 (0.58–0.79) |
| Favorable | 0.41 (0.32–0.53) | 0.43 (0.34–0.55) |

CI confidence interval, CVD cardiovascular disease, HR hazard ratio.

*Model adjusted for age, sex, education, residence, alcohol consumption, income and air pollutants (PM_1_, PM_2.5_, PM_10_ and NO_2_).

**Table S5.** Joint effects of lifestyle and air pollutant exposure on the incidence of CVD

|  |  | PM1 | PM_2.5_ | PM_10_ | NO_2_ | O_3_ |
| --- | --- | --- | --- | --- | --- | --- |
| Q1 | Favorable | 0.41 (0.26–0.65) | 0.36 (0.23–0.59) | 0.31 (0.19–0.51) | 0.28 (0.17–0.47) | 0.50 (0.32–0.78) |
|  | Intermediate | 0.54 (0.44–0.67) | 0.64 (0.52–0.79) | 0.43 (0.34–0.54) | 0.49 (0.39–0.61) | 0.62 (0.50–0.76) |
|  | Unfavorable | 0.49 (0.29–0.80) | 0.63 (0.40–0.99) | 0.42 (0.25–0.70) | 0.38 (0.21–0.66) | 0.80 (0.55–1.17) |
| Q2–Q5 | Favorable | 0.37 (0.28–0.48) | 0.39 (0.30–0.52) | 0.38 (0.29–0.50) | 0.40 (0.30–0.52) | 0.37 (0.28–0.49) |
|  | Intermediate | 0.61 (0.52–0.72) | 0.61 (0.51–0.72) | 0.62 (0.53–0.74) | 0.61 (0.52–0.72) | 0.63 (0.53–0.75) |
|  | Unfavorable | Reference | Reference | Reference | Reference | Reference |

Data presented as HR (95% CI).

CI confidence interval, CVD cardiovascular disease, HR hazard ratio, NO_2_ nitrogen dioxide, O_3_ ozone, PM_1_ particulate matter with an aerodynamic diameter less than 1 μm, PM_2.5_ particulate matter with an aerodynamic diameter less than 2.5 μm, PM_10_ particulate matter with an aerodynamic diameter <10 μm.

**Table S6.** The HRs (95% CIs) of incident CVD associated with each lifestyle factor at different levels of air pollutant exposure

|  | All | PM_1_ | | PM_2.5_ | | PM10 | | NO_2_ | | O_3_ | |
| --- | --- | --- | --- | --- | --- | --- | --- | --- | --- | --- | --- |
|  |  | Q1 | Q2–Q5 | Q1 | Q2–Q5 | Q1 | Q2–Q5 | Q1 | Q2–Q5 | Q1 | Q2–Q5 |
| Target blood pressure | 0.65 (0.57–0.74) | 0.53 (0.38–0.74) | 0.67 (0.59–0.78) | 0.49 (0.36–0.68) | 0.69 (0.60–0.80) | 0.46 (0.32–0.67) | 0.68 (0.60–0.79) | 0.50 (0.35–0.71) | 0.69 (0.60–0.79) | 0.65 (0.49–0.87) | 0.65 (0.56–0.75) |
| Target blood glucose | 0.87 (0.77–0.98) | 1.26 (0.95–1.67) | 0.81 (0.71–0.92) | 1.00 (0.76–1.31) | 0.84 (0.74–0.96) | 1.09 (0.80–1.48) | 0.86 (0.75–0.97) | 1.15 (0.86–1.54) | 0.84 (0.74–0.96) | 0.93 (0.71–1.22) | 0.86 (0.76–0.98) |
| Target total cholesterol | 0.89 (0.79–0.99) | 1.08 (0.82–1.44) | 0.84 (0.74–0.96) | 0.94 (0.72–1.24) | 0.87 (0.77–0.99) | 1.09 (0.79–1.49) | 0.83 (0.74–0.95) | 0.92 (0.68–1.24) | 0.87 (0.77–0.99) | 1.03 (0.79–1.34) | 0.85 (0.75–0.97) |
| Nonoverweight | 0.60 (0.54–0.68) | 0.62 (0.45–0.87) | 0.60 (0.53–0.69) | 0.63 (0.47–0.85) | 0.60 (0.53–0.68) | 0.67 (0.46–0.98) | 0.61 (0.54–0.70) | 0.75 (0.52–1.09) | 0.60 (0.53–0.68) | 0.73 (0.55–0.97) | 0.58 (0.51–0.66) |
| Never smoking | 0.80 (0.68–0.94) | 0.83 (0.55–1.25) | 0.80 (0.67–0.96) | 0.86 (0.58–1.27) | 0.79 (0.66–0.95) | 0.81 (0.53–1.23) | 0.79 (0.66–0.95) | 0.88 (0.58–1.34) | 0.79 (0.66–0.94) | 0.61 (0.42–0.89) | 0.85 (0.71–1.03) |
| Physical activity | 0.87 (0.77–0.99) | 0.96 (0.70–1.31) | 0.86 (0.74–0.99) | 0.96 (0.70–1.30) | 0.85 (0.74–0.99) | 0.97 (0.69–1.37) | 0.86 (0.74–0.99) | 1.13 (0.82–1.56) | 0.83 (0.72–0.96) | 0.93 (0.69–1.25) | 0.86 (0.75–1.00) |
| Each 1-point in lifestyle score | 0.81 (0.77–0.85) | 0.89 (0.79–1.00) | 0.80 (0.76–0.84) | 0.83 (0.74–0.93) | 0.81 (0.77–0.85) | 0.86 (0.75–0.98) | 0.81 (0.77–0.85) | 0.90 (0.79–1.01) | 0.80 (0.76–0.85) | 0.85 (0.76–0.95) | 0.80 (0.76–0.85) |

CI confidence interval, CVD cardiovascular disease, HR hazard ratio, NO_2_ nitrogen dioxide, O_3_ ozone, PM_1_ particulate matter with an aerodynamic diameter less than 1 μm, PM_2.5_ particulate matter with an aerodynamic diameter less than 2.5 μm, PM_10_ particulate matter with an aerodynamic diameter <10 μm.

**Table S7.** Subgroup analysis of the additive interactions analysis of the effect of dichotomized lifestyle on the association between ambient air pollutant exposure and CVD in high air pollutant exposure levels (Q2–Q5)

|  | n | HR (95% CI)* | p for interaction |
| --- | --- | --- | --- |
| PM1 |  |  |  |
| Age |  |  | 0.210 |
| < 65 years old | 4360 | 0.53 (0.43–0.64) |  |
| ≥ 65 years old | 1256 | 0.70 (0.51–0.95) |  |
| Sex |  |  | 0.021 |
| Male | 2594 | 0.48 (0.39–0.60) |  |
| Female | 3022 | 0.72 (0.56–0.93) |  |
| PM_2.5_ |  |  |  |
| Age |  |  | 0.289 |
| < 65 years old | 4301 | 0.54 (0.44–0.66) |  |
| ≥ 65 years old | 1293 | 0.69 (0.51–0.94) |  |
| Sex |  |  | 0.038 |
| Male | 2585 | 0.50 (0.40–0.62) |  |
| Female | 3009 | 0.72 (0.55–0.93) |  |
| PM10 |  |  |  |
| Age |  |  | 0.528 |
| < 65 years old | 4350 | 0.56 (0.46–0.68) |  |
| ≥ 65 years old | 1238 | 0.67 (0.49–0.91) |  |
| Sex |  |  | 0.041 |
| Male | 2589 | 0.50 (0.41–0.63) |  |
| Female | 2999 | 0.72 (0.56–0.93) |  |
| NO_2_ |  |  |  |
| Age |  |  | 0.434 |
| < 65 years old | 4374 | 0.55 (0.45–0.67) |  |
| ≥ 65 years old | 1225 | 0.67 (0.49–0.91) |  |
| Sex |  |  | 0.056 |
| Male | 2605 | 0.50 (0.40–0.62) |  |
| Female | 2994 | 0.70 (0.54–0.90) |  |
| O_3_ |  |  |  |
| Age |  |  | 0.128 |
| < 65 years old | 4416 | 0.53 (0.44–0.65) |  |
| ≥ 65 years old | 1214 | 0.75 (0.54–1.05) |  |
| Sex |  |  | 0.021 |
| Male | 2575 | 0.50 (0.40–0.62) |  |
| Female | 3055 | 0.76 (0.58–0.99) |  |

*HR (95% CI) comparing those with intermediate or favorable lifestyle versus those with unfavorable lifestyle in high levels of air pollution (Q2–Q5) are shown.

CI confidence interval, CVD cardiovascular disease, HR hazard ratio, NO_2_ nitrogen dioxide, O_3_ ozone, PM_1_ particulate matter with an aerodynamic diameter less than 1 μm, PM_2.5_ particulate matter with an aerodynamic diameter less than 2.5 μm, PM_10_ particulate matter with an aerodynamic diameter <10 μm.

**Table S8.** The HRs (95% CIs) of associations between air pollutant exposure (per 10 μg/m^3^ increase) and incident CVD, and the mediation effect of lifestyle categories on air pollution and CVD in different sensitivity analysis models

|  | Unfavorable | Intermediate | Favorable | Mediation proportion (%) (95% CI) |
| --- | --- | --- | --- | --- |
| PM_1_ |  |  |  |  |
| Model 4 | 1.17 (1.05–1.30) | 1.08 (1.03–1.14) | 0.99 (0.84–1.16) | 7.4 (3.2–15.6) |
| Model 5 | 1.18 (1.06–1.32) | 1.08 (1.03–1.13) | 0.98 (0.84–1.15) | 7.9 (3.5–16.7) |
| Model 6 | 1.69 (1.46–1.97) | 1.44 (1.34–1.54) | 1.24 (0.99–1.54) | 4.0 (2.3–6.4) |
| Model 7 | 1.20 (1.07–1.34) | 1.10 (1.05–1.16) | 1.00 (0.85–1.18) | 7.4 (3.7–14.1) |
| Model 8 | 1.09 (0.99–1.19) | 1.10 (1.04–1.16) | 0.99 (0.87–1.13) | 6.8 (2.3–15.9) |
| Model 9 | 1.19 (1.01–1.40) | 1.09 (1.04–1.15) | 1.04 (0.94–1.16) | 5.1 (1.2–12.2) |
| Model 10 | 1.22 (1.08–1.37) | 1.09 (1.04–1.15) | 0.96 (0.81–1.14) | 7.0 (3.1–14.9) |
| Model 11 | 1.22 (1.08–1.37) | 1.10 (1.04–1.16) | 0.97 (0.82–1.15) | 6.4 (2.7–13.4) |
| Model 12 | 1.17 (1.04–1.33) | 1.10 (1.04–1.16) | 1.01 (0.85–1.19) | 7.26 (3.13–15.8) |
| PM_2.5_ |  |  |  |  |
| Model 4 | 1.12 (1.02–1.24) | 1.03 (0.98–1.07) | 0.96 (0.84–1.10) | 12.8 (4.8–49.6) |
| Model 5 | 1.13 (1.03–1.25) | 1.03 (0.98–1.07) | 0.96 (0.84–1.10) | 13.9 (4.9–60.3) |
| Model 6 | 1.31 (1.18–1.45) | 1.18 (1.12–1.24) | 1.08 (0.93–1.26) | 5.7 (3.3–9.4) |
| Model 7 | 1.15 (1.04–1.27) | 1.04 (1.00–1.09) | 0.99 (0.86–1.12) | 11.2 (5.7–31.1) |
| Model 8 | 1.05 (0.97–1.14) | 1.04 (1.00–1.09) | 0.98 (0.88–1.09) | 11.2 (3.0–48.4) |
| Model 9 | 1.17 (1.01–1.36) | 1.03 (0.99–1.08) | 1.00 (0.92–1.10) | 9.7 (1.9–48.7) |
| Model 10 | 1.16 (1.05–1.29) | 1.04 (1.00–1.08) | 0.92 (0.80–1.06) | 12.3 (4.5–48.3) |
| Model 11 | 1.15 (1.04–1.28) | 1.04 (1.00–1.09) | 0.96 (0.84–1.11) | 10.5 (4.2–31.9) |
| Model 12 | 1.13 (1.02–1.26) | 1.04 (0.99–1.09) | 0.98 (0.85–1.13) | 12.4 (4.5–51.9) |
| PM_10_ |  |  |  |  |
| Model 4 | 1.09 (1.04–1.15) | 1.05 (1.02–1.07) | 1.01 (0.94–1.09) | 7.3 (3.8–13.3) |
| Model 5 | 1.09 (1.04–1.15) | 1.04 (1.02–1.07) | 1.01 (0.94–1.09) | 7.8 (4.2–14.2) |
| Model 6 | 1.17 (1.09–1.25) | 1.13 (1.09–1.16) | 1.11 (1.01–1.22) | 2.8 (1.7–4.3) |
| Model 7 | 1.10 (1.04–1.16) | 1.05 (1.03–1.08) | 1.02 (0.95–1.10) | 7.3 (3.9–12.6) |
| Model 8 | 1.05 (1.00–1.10) | 1.05 (1.03–1.08) | 1.02 (0.96–1.08) | 7.3 (3.7–13.4) |
| Model 9 | 1.10 (1.02–1.19) | 1.05 (1.03–1.08) | 1.03 (0.98–1.09) | 5.3 (2.3–10.2) |
| Model 10 | 1.11 (1.05–1.17) | 1.05 (1.02–1.08) | 0.98 (0.90–1.06) | 7.0 (3.4–12.9) |
| Model 11 | 1.11 (1.05–1.17) | 1.05 (1.03–1.08) | 1.02 (0.94–1.10) | 6.2 (3.1–11.2) |
| Model 12 | 1.09 (1.03–1.15) | 1.05 (1.02–1.07) | 1.03 (0.95–1.11) | 7.3 (3.4–14.1) |
| NO_2_ |  |  |  |  |
| Model 4 | 1.25 (1.09–1.44) | 1.08 (1.01–1.15) | 0.99 (0.80–1.21) | 11.2 (6.0–22.9) |
| Model 5 | 1.26 (1.10–1.45) | 1.09 (1.02–1.16) | 0.99 (0.80–1.21) | 11.6 (6.1–23.7) |
| Model 6 | 1.37 (1.28–1.46) | 1.27 (1.23–1.31) | 1.23 (1.11–1.36) | 17.2 (7.8–70.5) |
| Model 7 | 1.25 (1.09–1.43) | 1.10 (1.03–1.17) | 1.00 (0.82–1.23) | 10.9 (5.9–20.2) |
| Model 8 | 1.13 (1.00–1.28) | 1.11 (1.04–1.18) | 1.00 (0.85–1.18) | 11.2 (5.7–23.2) |
| Model 9 | 1.35 (1.09–1.66) | 1.10 (1.03–1.18) | 1.04 (0.91–1.19) | 8.9 (4.1–19.7) |
| Model 10 | 1.32 (1.14–1.53) | 1.10 (1.03–1.17) | 0.92 (0.74–1.15) | 10.2 (5.0–21.8) |
| Model 11 | 1.31 (1.13–1.52) | 1.11 (1.04–1.19) | 1.01 (0.81–1.25) | 9.2 (4.8–17.6) |
| Model 12 | 1.27 (1.09–1.49) | 1.08 (1.01–1.16) | 1.02 (0.82–1.28) | 11.2 (5.4–26.5) |
| O_3_ |  |  |  |  |
| Model 4 | 1.23 (0.99–1.54) | 1.01 (0.91–1.13) | 0.88 (0.63–1.24) | - |
| Model 5 | 1.25 (1.00–1.56) | 1.00 (0.90–1.11) | 0.88 (0.63–1.24) | - |
| Model 6 | 1.11 (0.92–1.36) | 0.98 (0.89–1.09) | 0.85 (0.62–1.18) | - |
| Model 7 | 1.31 (1.03–1.66) | 1.04 (0.93–1.16) | 0.84 (0.59–1.21) | - |
| Model 8 | 1.11 (0.92–1.35) | 1.02 (0.92–1.14) | 0.96 (0.73–1.26) |  |
| Model 9 | 1.38 (0.99–1.92) | 1.03 (0.92–1.14) | 1.01 (0.81–1.27) | - |
| Model 10 | 1.34 (1.07–1.70) | 1.04 (0.93–1.16) | 0.86 (0.60–1.22) | - |
| Model 11 | 1.34 (1.05–1.70) | 1.01 (0.90–1.13) | 0.84 (0.58–1.21) | - |
| Model 12 | 1.28 (0.99–1.65) | 1.08 (0.96–1.21) | 0.95 (0.65–1.39) |  |

Model 4: adjusted for age, sex, education, residence, alcohol consumption, income, and depressive symptoms (n=6881).

Model 5: adjusted for age, sex, education, residence, alcohol consumption, income, and solid fuel use (n=6947).

Model 6: the associations between time-varying ambient air pollutant exposure (per 10 μg/m^3^) and CVD in different lifestyle categories.

Model 7: the associations between three-year ambient air pollutant exposure (per 10 μg/m^3^) and CVD in different lifestyle categories.

Model 8: the associations between ambient air pollutant exposure (per 10 μg/m^3^) and CVD in new assignment of lifestyle categories.

Model 9: the associations between ambient air pollutant exposure (per 10 μg/m^3^) and CVD in new lifestyle categories considering sleep (n=6951).

Model 10: excluded those with missing baseline information (n=6235).

Model 11: excluded those with chronic lung disease (n= 5689).

Model 12: excluded those who did not change residence (n= 5519).

CI confidence interval, CVD cardiovascular disease, HR hazard ratio, NO_2_ nitrogen dioxide, O_3_ ozone, PM_1_ particulate matter with an aerodynamic diameter less than 1 μm, PM_2.5_ particulate matter with an aerodynamic diameter less than 2.5 μm, PM_10_ particulate matter with an aerodynamic diameter <10 μm.

**Table S9.** The HRs (95% CIs) of the associations between ambient air pollutant exposure (per 10 μg/m^3^ increase) and CVD in different lifestyle categories in different sensitivity analysis models

|  | Model 4  (HR [95% CI]) | Model 5  (HR [95% CI]) | Model 6  (HR [95% CI]) | Model 7  (HR [95% CI]) | Model 8  (HR [95% CI]) | Model 9  (HR [95% CI]) | Model 10  (HR [95% CI]) | Model 11  (HR [95% CI]) | Model 12  (HR [95% CI]) |
| --- | --- | --- | --- | --- | --- | --- | --- | --- | --- |
| PM_1_ |  |  |  |  |  |  |  |  |  |
| Q1 |  |  |  |  |  |  |  |  |  |
| Unfavorable | Reference | Reference | Reference | Reference | Reference | Reference | Reference | Reference | Reference |
| Intermediate | 1.03  (0.62–1.71) | 1.20  (0.70–2.06) | 0.86  (0.53–1.40) | 0.95  (0.58–1.54) | 0.80  (0.52–1.23) | 0.97  (0.49–1.90) | 1.30  (0.73–2.32) | 1.21  (0.70–2.11) | 1.14  (0.64–2.03) |
| Favorable | 0.79  (0.41–1.53) | 0.91  (0.46–1.82) | 0.71  (0.37–1.40) | 0.58  (0.30–1.13) | 0.63  (0.35–1.12) | 0.68  (0.33–1.42) | 1.14  (0.56–2.34) | 1.01  (0.50–2.05) | 0.96  (0.46–2.01) |
| Q2–Q5 |  |  |  |  |  |  |  |  |  |
| Unfavorable | Reference | Reference | Reference | Reference | Reference | Reference | Reference | Reference | Reference |
| Intermediate | 0.59  (0.50–0.70) | 0.60  (0.51–0.71) | 0.63  (0.53–0.74) | 0.62  (0.52–0.73) | 0.64  (0.55–0.74) | 0.68  (0.54–0.86) | 0.61  (0.52–0.73) | 0.64  (0.53–0.76) | 0.63  (0.52–0.76) |
| Favorable | 0.35  (0.26–0.46) | 0.36  (0.28–0.48) | 0.37  (0.28–0.49) | 0.39  (0.29–0.51) | 0.41  (0.32–0.51) | 0.44  (0.33–0.57) | 0.36  (0.27–0.48) | 0.36  (0.28–0.49) | 0.38  (0.28–0.52) |
| PM_2.5_ |  |  |  |  |  |  |  |  |  |
| Q1 |  |  |  |  |  |  |  |  |  |
| Unfavorable | Reference | Reference | Reference | Reference | Reference | Reference | Reference | Reference | Reference |
| Intermediate | 0.97  (0.61–1.55) | 1.20  (0.70–2.06) | 0.97  (0.62–1.55) | 1.08  (0.66–1.77) | 0.81  (0.56–1.18) | 1.28  (0.63–2.60) | 1.09  (0.65–1.81) | 1.06  (0.70–1.75) | 1.11  (0.65–1.90) |
| Favorable | 0.55  (0.29–1.04) | 0.91  (0.46–1.82) | 0.61  (0.62–1.55) | 0.66  (0.35–1.27) | 0.50  (0.29–0.84) | 0.69  (0.32–1.50) | 0.70  (0.36–1.36) | 0.65  (0.33–1.27) | 0.70  (0.34–1.42) |
| Q2–Q5 |  |  |  |  |  |  |  |  |  |
| Unfavorable | Reference | Reference | Reference | Reference | Reference | Reference | Reference | Reference | Reference |
| Intermediate | 0.59  (0.50–0.70) | 0.60  (0.51–0.71) | 0.61  (0.52–0.72) | 0.61  (0.51–0.71) | 0.63  (0.54–0.74) | 0.65  (0.52–0.82) | 0.62  (0.52–0.73) | 0.64  (0.53–0.77) | 0.63  (0.52–0.76) |
| Favorable | 0.38  (0.28–0.50) | 0.36  (0.28–0.48) | 0.39  (0.29–0.51) | 0.38  (0.29–0.50) | 0.42  (0.34–0.54) | 0.45  (0.34–0.58) | 0.39  (0.29–0.53) | 0.39  (0.29–0.53) | 0.41  (0.30–0.55) |
| PM_10_ |  |  |  |  |  |  |  |  |  |
| Q1 |  |  |  |  |  |  |  |  |  |
| Unfavorable | Reference | Reference | Reference | Reference | Reference | Reference | Reference | Reference | Reference |
| Intermediate | 0.99  (0.58–1.70) | 1.03  (0.60–1.76) | 1.23  (0.62–2.45) | 1.01  (0.60–1.67) | 0.80  (0.52–1.23) | 0.83  (0.42–1.63) | 1.03  (0.58–1.83) | 1.14  (0.63–2.08) | 1.15  (0.62–2.15) |
| Favorable | 0.76  (0.37–1.53) | 0.81  (0.40–1.63) | 1.16  (0.50–2.70) | 0.74  (0.37–1.47) | 0.63  (0.35–1.12) | 0.58  (0.28–1.21) | 0.96  (0.46–2.00) | 0.99  (0.47–2.11) | 0.93  (0.42–2.05) |
| Q2–Q5 |  |  |  |  |  |  |  |  |  |
| Unfavorable | Reference | Reference | Reference | Reference | Reference | Reference | Reference | Reference | Reference |
| Intermediate | 0.60  (0.51–0.71) | 0.62  (0.53–0.73) | 0.63  (0.54–0.74) | 0.62  (0.53–0.73) | 0.64  (0.55–0.74) | 0.71  (0.56–0.89) | 0.64  (0.54–0.76) | 0.65  (0.55–0.78) | 0.64  (0.53–0.77) |
| Favorable | 0.36  (0.28–0.48) | 0.38  (0.29–0.50) | 0.38  (0.29–0.49) | 0.38  (0.29–0.50) | 0.41  (0.32–0.51) | 0.45  (0.35–0.59) | 0.38  (0.29–0.51) | 0.38  (0.28–0.51) | 0.39  (0.29–0.53) |
| NO_2_ |  |  |  |  |  |  |  |  |  |
| Q1 |  |  |  |  |  |  |  |  |  |
| Unfavorable | Reference | Reference | Reference | Reference | Reference | Reference | Reference | Reference | Reference |
| Intermediate | 1.25  (0.70–2.21) | 1.29  (0.73–2.29) | 0.79  (0.51–1.22) | 1.00  (0.57–1.73) | 0.93  (0.60–1.43) | 1.46  (0.59–3.57) | 1.16  (0.65–2.05) | 1.66  (0.84–3.27) | 1.27  (0.68–2.37) |
| Favorable | 0.74  (0.36–1.55) | 0.79  (0.38–1.64) | 0.52  (0.29–0.93) | 0.66  (0.32–1.35) | 0.67  (0.38–1.18) | 1.13  (0.44–2.88) | 0.80  (0.38–1.68) | 1.05  (0.46–2.39) | 0.65  (0.28–1.50) |
| Q2–Q5 |  |  |  |  |  |  |  |  |  |
| Unfavorable | Reference | Reference | Reference | Reference | Reference | Reference | Reference | Reference | Reference |
| Intermediate | 0.59  (0.50–0.70) | 0.61  (0.52–0.72) | 0.63  (0.53–0.74) | 0.64 (0.54–0.75) | 0.63  (0.54–0.73) | 0.68  (0.54–0.85) | 0.63  (0.53–0.75) | 0.64  (0.53–0.76) | 0.63  (0.53–0.76) |
| Favorable | 0.38  (0.29–0.49) | 0.39  (0.30–0.52) | 0.39  (0.30–0.52) | 0.41  (0.31–0.53) | 0.41  (0.33–0.52) | 0.43  (0.33–0.56) | 0.40  (0.30–0.53) | 0.39  (0.29–0.53) | 0.43  (0.32–0.58) |
| O_3_ |  |  |  |  |  |  |  |  |  |
| Q1 |  |  |  |  |  |  |  |  |  |
| Unfavorable | Reference | Reference | Reference | Reference | Reference | Reference | Reference | Reference | Reference |
| Intermediate | 0.74  (0.50–1.08) | 0.76  (0.52–1.12) | 0.83  (0.56–1.23) | 0.80  (0.54–1.20) | 0.66  (0.48–0.93) | 0.90  (0.53–1.53) | 0.90  (0.59–1.36) | 0.87  (0.57–1.33) | 0.71  (0.46–1.09) |
| Favorable | 0.58  (0.34–1.01) | 0.62  (0.36–1.06) | 0.54  (0.30–1.00) | 0.63  (0.35–1.13) | 0.52  (0.33–0.84) | 0.59  (0.32–1.08) | 0.75  (0.42–1.35) | 0.72  (0.40–1.31) | 0.61  (0.33–1.13) |
| Q2–Q5 |  |  |  |  |  |  |  |  |  |
| Unfavorable | Reference | Reference | Reference | Reference | Reference | Reference | Reference | Reference | Reference |
| Intermediate | 0.61  (0.52–0.73) | 0.63  (0.53–0.75) | 0.62  (0.52–0.74) | 0.63  (0.53–0.75) | 0.66  (0.56–0.77) | 0.68  (0.53–0.86) | 0.62  (0.52–0.75) | 0.66  (0.54–0.79) | 0.67  (0.55–0.81) |
| Favorable | 0.35  (0.27–0.47) | 0.37  (0.28–0.49) | 0.39  (0.29–0.51) | 0.38  (0.28–0.50) | 0.41  (0.32–0.52) | 0.34  (0.33–0.58) | 0.37  (0.27–0.50) | 0.37  (0.27–0.50) | 0.40  (0.29–0.54) |

Model 4: adjusted for age, sex, education, residence, alcohol consumption, income, and depressive symptoms (n=6881).

Model 5: adjusted for age, sex, education, residence, alcohol consumption, income, and solid fuel use (n=6947).

Model 6: the associations between time-varying ambient air pollutant exposure (per 10 μg/m^3^) and CVD in different lifestyle categories.

Model 7: the associations between three-year ambient air pollutant exposure (per 10 μg/m^3^) and CVD in different lifestyle categories.

Model 8: the associations between ambient air pollutant exposure (per 10 μg/m^3^) and CVD in new assignment of lifestyle categories.

Model 9: the associations between ambient air pollutant exposure (per 10 μg/m^3^) and CVD in new lifestyle categories considering sleep (n=6951).

Model 10: excluding those with missing baseline information (n=6235).

Model 11: excluding those with chronic lung disease (n= 5689).

Model 12: excluding those who did not change residence (n= 5519).

CI confidence interval, CVD cardiovascular disease, HR hazard ratio, NO_2_ nitrogen dioxide, O_3_ ozone, PM_1_ particulate matter with an aerodynamic diameter less than 1 μm, PM_2.5_ particulate matter with an aerodynamic diameter less than 2.5 μm, PM_10_ particulate matter with an aerodynamic diameter <10 μm.

**Table S10.** Multiplicative and additive interaction analysis of the effect of dichotomized lifestyle on the association between time-varying ambient air pollutant exposure and CVD

|  | HR (95% CI) | p for multiplicative interaction | RERI (95% CI) |
| --- | --- | --- | --- |
| PM_1_ |  | 0.096 | -1.31 (-1.92–-0.70) |
| Q1 |  |  |  |
| Unfavorable | Reference |  |  |
| Intermediate & Favorable | 0.90 (0.56–1.47) |  |  |
| Q2–Q5 |  |  |  |
| Unfavorable | Reference |  |  |
| Intermediate & Favorable | 0.59 (0.50–0.69) |  |  |
| PM_2.5_ |  | 0.021 | -0.80 (-1.30–-0.30) |
| Q1 |  |  |  |
| Unfavorable | Reference |  |  |
| Intermediate & Favorable | 1.02 (0.62–1.67) |  |  |
| Q2–Q5 |  |  |  |
| Unfavorable | Reference |  |  |
| Intermediate & Favorable | 0.58 (0.49–0.68) |  |  |
| PM_10_ |  | 0.083 | -3.61 (-4.80–-2.43) |
| Q1 |  |  |  |
| Unfavorable | Reference |  |  |
| Intermediate & Favorable | 0.97 (0.59–1.61) |  |  |
| Q2–Q5 |  |  |  |
| Unfavorable | Reference |  |  |
| Intermediate & Favorable | 0.59 (0.50–0.69) |  |  |
| NO_2_ |  | 0.166 | -0.63 (-1.09–-0.18) |
| Q1 |  |  |  |
| Unfavorable | Reference |  |  |
| Intermediate & Favorable | 0.95 (0.55–1.66) |  |  |
| Q2–Q5 |  |  |  |
| Unfavorable | Reference |  |  |
| Intermediate & Favorable | 0.61 (0.52–0.71) |  |  |
| O_3_ |  | 0.205 | -0.99 (-1.52–-0.46) |
| Q1 |  |  |  |
| Unfavorable | Reference |  |  |
| Intermediate & Favorable | 0.78 (0.53–1.17) |  |  |
| Q2–Q5 |  |  |  |
| Unfavorable | Reference |  |  |
| Intermediate & Favorable | 0.60 (0.50–0.71) |  |  |

CI confidence interval, CVD cardiovascular disease, HR hazard ratio, NO_2_ nitrogen dioxide, O_3_ ozone, PM_1_ particulate matter with an aerodynamic diameter less than 1 μm, PM_2.5_ particulate matter with an aerodynamic diameter less than 2.5 μm, PM_10_ particulate matter with an aerodynamic diameter <10 μm, RERI relative excess risk due to interaction.

**Table S11.** Multiplicative and additive interaction analysis of the effect of dichotomized lifestyle on the association between three years of ambient air pollutant exposure and CVD

|  | HR (95% CI) | p for multiplicative interaction | RERI (95% CI) |
| --- | --- | --- | --- |
| PM_1_ |  | 0.087 | -1.09 (-1.65–-0.53) |
| Q1 |  |  |  |
| Unfavorable | Reference |  |  |
| Intermediate & Favorable | 0.84 (0.51–1.36) |  |  |
| Q2–Q5 |  |  |  |
| Unfavorable | Reference |  |  |
| Intermediate & Favorable | 0.57 (0.49–0.68) |  |  |
| PM_2.5_ |  | 0.079 | -0.83 (-1.33–-0.33) |
| Q1 |  |  |  |
| Unfavorable | Reference |  |  |
| Intermediate & Favorable | 0.99 (0.61–1.63) |  |  |
| Q2–Q5 |  |  |  |
| Unfavorable | Reference |  |  |
| Intermediate & Favorable | 0.56 (0.47–0.66) |  |  |
| PM_10_ |  | 0.664 | -1.71 (-2.41–-1.01) |
| Q1 |  |  |  |
| Unfavorable | Reference |  |  |
| Intermediate & Favorable | 0.94 (0.56–1.56) |  |  |
| Q2–Q5 |  |  |  |
| Unfavorable | Reference |  |  |
| Intermediate & Favorable | 0.57 (0.49–0.67) |  |  |
| NO_2_ |  | 0.083 | -1.68 (-2.36–-0.99) |
| Q1 |  |  |  |
| Unfavorable | Reference |  |  |
| Intermediate & Favorable | 0.90 (0.51–1.56) |  |  |
| Q2–Q5 |  |  |  |
| Unfavorable | Reference |  |  |
| Intermediate & Favorable | 0.59 (0.50–0.70) |  |  |
| O_3_ |  | 0.242 | -0.94 (-1.47–-0.42) |
| Q1 |  |  |  |
| Unfavorable | Reference |  |  |
| Intermediate & Favorable | 0.74 (0.50–1.11) |  |  |
| Q2–Q5 |  |  |  |
| Unfavorable | Reference |  |  |
| Intermediate & Favorable | 0.58 (0.49–0.69) |  |  |

CI confidence interval, CVD cardiovascular disease, HR hazard ratio, NO_2_ nitrogen dioxide, O_3_ ozone, PM_1_ particulate matter with an aerodynamic diameter less than 1 μm, PM_2.5_ particulate matter with an aerodynamic diameter less than 2.5 μm, PM_10_ particulate matter with an aerodynamic diameter <10 μm, RERI relative excess risk due to interaction.

**Table S12.** Multiplicative and additive interaction analysis of the effect of dichotomized lifestyle considering nighttime sleep duration on the association between ambient air pollutant exposure and CVD

|  | HR (95% CI) | p for multiplicative interaction | RERI (95% CI) |
| --- | --- | --- | --- |
| PM_1_ |  | 0.212 | -0.94 (-1.54–-0.35) |
| Q1 |  |  |  |
| Unfavorable | Reference |  |  |
| Intermediate & Favorable | 0.90 (0.46–1.77) |  |  |
| Q2–Q5 |  |  |  |
| Unfavorable | Reference |  |  |
| Intermediate & Favorable | 0.62 (0.49–0.78) |  |  |
| PM_2.5_ |  | 0.079 | -0.56 (-1.07–-0.06) |
| Q1 |  |  |  |
| Unfavorable | Reference |  |  |
| Intermediate & Favorable | 1.12 (0.55–2.29) |  |  |
| Q2–Q5 |  |  |  |
| Unfavorable | Reference |  |  |
| Intermediate & Favorable | 0.60 (0.48–0.76) |  |  |
| PM_10_ |  | 0.664 | -1.77 (-2.56–-0.98) |
| Q1 |  |  |  |
| Unfavorable | Reference |  |  |
| Intermediate & Favorable | 0.75 (0.39–1.49) |  |  |
| Q2–Q5 |  |  |  |
| Unfavorable | Reference |  |  |
| Intermediate & Favorable | 0.65 (0.51–0.81) |  |  |
| NO_2_ |  | 0.083 | -1.43 (-2.13–-0.73) |
| Q1 |  |  |  |
| Unfavorable | Reference |  |  |
| Intermediate & Favorable | 1.37 (0.56–3.34) |  |  |
| Q2–Q5 |  |  |  |
| Unfavorable | Reference |  |  |
| Intermediate & Favorable | 0.62 (0.49–0.77) |  |  |
| O_3_ |  | 0.242 | -0.58 (-1.10–-0.07) |
| Q1 |  |  |  |
| Unfavorable | Reference |  |  |
| Intermediate & Favorable | 0.83 (0.49–1.40) |  |  |
| Q2–Q5 |  |  |  |
| Unfavorable | Reference |  |  |
| Intermediate & Favorable | 0.62 (0.49–0.78) |  |  |

CI confidence interval, CVD cardiovascular disease, HR hazard ratio, NO_2_ nitrogen dioxide, O_3_ ozone, PM_1_ particulate matter with an aerodynamic diameter less than 1 μm, PM_2.5_ particulate matter with an aerodynamic diameter less than 2.5 μm, PM_10_ particulate matter with an aerodynamic diameter <10 μm, RERI relative excess risk due to interaction.

**Table S13.** Multiplicative and additive interaction analysis of the effect of dichotomized lifestyle considering new assignment of lifestyle categories on the association between ambient air pollutant exposure and CVD

|  | HR (95% CI) | p for multiplicative interaction | RERI (95% CI) |
| --- | --- | --- | --- |
| PM_1_ |  | 0.013 | -0.92 (-2.04–0.21) |
| Q1 |  |  |  |
| Unfavorable | Reference |  |  |
| Intermediate & Favorable | 0.98 (0.65–1.48) |  |  |
| Q2–Q5 |  |  |  |
| Unfavorable | Reference |  |  |
| Intermediate & Favorable | 0.57 (0.49–0.66) |  |  |
| PM_2.5_ |  | 0.176 | -0.01 (-0.71–0.69) |
| Q1 |  |  |  |
| Unfavorable | Reference |  |  |
| Intermediate & Favorable | 0.77 (0.51–1.18) |  |  |
| Q2–Q5 |  |  |  |
| Unfavorable | Reference |  |  |
| Intermediate & Favorable | 0.59 (0.51–0.69) |  |  |
| PM_10_ |  | 0.279 | -0.97 (-2.13–0.19) |
| Q1 |  |  |  |
| Unfavorable | Reference |  |  |
| Intermediate & Favorable | 0.59 (0.51–0.69) |  |  |
| Q2–Q5 |  |  |  |
| Unfavorable | Reference |  |  |
| Intermediate & Favorable | 0.65 (0.51–0.81) |  |  |
| NO_2_ |  | 0.109 | -0.96 (-2.16–0.24) |
| Q1 |  |  |  |
| Unfavorable | Reference |  |  |
| Intermediate & Favorable | 0.88 (0.57–1.35) |  |  |
| Q2–Q5 |  |  |  |
| Unfavorable | Reference |  |  |
| Intermediate & Favorable | 0.59 (0.51–0.69) |  |  |
| O_3_ |  | 0.702 | 0.29 (-0.23–0.81) |
| Q1 |  |  |  |
| Unfavorable | Reference |  |  |
| Intermediate & Favorable | 0.64 (0.46–0.88) |  |  |
| Q2–Q5 |  |  |  |
| Unfavorable | Reference |  |  |
| Intermediate & Favorable | 0.61 (0.52–0.72) |  |  |

CI confidence interval, CVD cardiovascular disease, HR hazard ratio, NO_2_ nitrogen dioxide, O_3_ ozone, PM_1_ particulate matter with an aerodynamic diameter less than 1 μm, PM_2.5_ particulate matter with an aerodynamic diameter less than 2.5 μm, PM_10_ particulate matter with an aerodynamic diameter <10 μm, RERI relative excess risk due to interaction.

**Table S14.** The subdistribution HRs (sHRs, 95% CI) of the associations between ambient air pollutant exposure (per 10 μg/m^3^) and CVD in different lifestyle categories

|  | Model 6 (sHR [95% CI])^a^ | Model 7 (sHR [95% CI])^b^ |
| --- | --- | --- |
| PM_1_ |  |  |
| Q1 |  |  |
| Unfavorable | Reference | Reference |
| Intermediate | 0.94 (0.66–1.33) | 1.22 (0.71–2.09) |
| Favorable | 0.79 (0.41–1.54) | 0.94 (0.47–1.87) |
| Q2–Q5 |  |  |
| Unfavorable | Reference | Reference |
| Intermediate | 0.68 (0.60–0.78) | 0.61 (0.51–0.71) |
| Favorable | 0.46 (0.37–0.58) | 0.37 (0.28–0.49) |
| PM_2.5_ |  |  |
| Q1 |  |  |
| Unfavorable | Reference | Reference |
| Intermediate | 0.92 (0.65–1.32) | 1.08 (0.67–1.75) |
| Favorable | 0.63 (0.38–1.02) | 0.60 (0.31–1.16) |
| Q2–Q5 |  |  |
| Unfavorable | Reference | Reference |
| Intermediate | 0.68 (0.60–0.78) | 0.61 (0.51–0.72) |
| Favorable | 0.48 (0.38–0.60) | 0.40 (0.30–0.53) |
| PM_10_ |  |  |
| Q1 |  |  |
| Unfavorable | Reference | Reference |
| Intermediate | 0.92 (0.64–1.33) | 1.02 (0.60–1.75) |
| Favorable | 0.70 (0.41–1.19) | 0.83 (0.41–1.68) |
| Q2–Q5 |  |  |
| Unfavorable | Reference | Reference |
| Intermediate | 0.69 (0.60–0.79) | 0.63 (0.53–0.74) |
| Favorable | 0.48 (0.38–0.59) | 0.38 (0.29–0.50) |
| NO_2_ |  |  |
| Q1 |  |  |
| Unfavorable | Reference | Reference |
| Intermediate | 1.18 (0.80–1.74) | 1.27 (0.72–2.25) |
| Favorable | 0.91 (0.54–1.53) | 0.77 (0.39–1.60) |
| Q2–Q5 |  |  |
| Unfavorable | Reference | Reference |
| Intermediate | 0.67 (0.59–0.77) | 0.62 (0.52–0.73) |
| Favorable | 0.46 (0.37–0.58) | 0.40 (0.30–0.53) |
| O_3_ |  |  |
| Q1 |  |  |
| Unfavorable | Reference | Reference |
| Intermediate | 0.78 (0.58–1.05) | 0.79 (0.54–1.15) |
| Favorable | 0.66 (0.43–1.02) | 0.65 (0.38–1.12) |
| Q2–Q5 |  |  |
| Unfavorable | Reference | Reference |
| Intermediate | 0.70 (0.61–0.81) | 0.63 (0.53–0.75) |
| Favorable | 0.46 (0.37–0.58) | 0.37 (0.28–0.49) |

Model adjusted for age, sex, education, residence, alcohol consumption and income.

CI confidence interval, CVD cardiovascular disease, HR hazard ratio, NO_2_ nitrogen dioxide, O_3_ ozone, PM_1_ particulate matter with an aerodynamic diameter less than 1 μm, PM_2.5_ particulate matter with an aerodynamic diameter less than 2.5 μm, PM_10_ particulate matter with an aerodynamic diameter <10 μm.

^a^Competing risk model considering death

^b^Death was replaced by absence of CVD

**Table S15.** Baseline characteristics of included and excluded participants

|  | Excluded  (n = 10705) | Included  (n = 7000) |
| --- | --- | --- |
| Age, years | 59.64 ± 10.93 | 58.43 ± 8.80 |
| Male | 5230 (48.86%) | 3247 (46.39%) |
| BMI, kg/m^2^ | 23.45 ± 4.08 | 23.49 ± 3.81 |
| Education |  |  |
| Primary school and below | 6896 (64.73%) | 5647 (70.47%) |
| Junior high school | 2224 (20.87%) | 1592 (19.87%) |
| High school and above | 1534 (14.40%) | 774 (9.66%) |
| Smoking | 4253 (40.16%) | 2678 (38.26%) |
| Alcohol consumption |  |  |
| More than once a month | 2554 (24.13%) | 1829 (26.13%) |
| Less than once a month | 809 (7.64%) | 575 (8.21%) |
| Never | 7220 (68.22%) | 4596 (65.66%) |
| SBP, mmHg | 130.17 ± 20.68 | 128.32 ± 20.84 |
| DBP, mmHg | 75.81 ± 11.79 | 74.98 ± 12.00 |
| Residence |  |  |
| Rural | 5875 (54.88%) | 4662 (66.60%) |
| Urban | 4830 (45.12%) | 2338 (33.40%) |
| Ambient air pollutants |  |  |
| PM_1_, μg/m^3^ | 40.27 ± 13.66 | 39.97 ± 13.80 |
| PM_10_, μg/m^3^ | 92.66 ± 28.15 | 93.44 ± 28.05 |
| PM_2.5_, μg/m^3^ | 52.15 ± 16.06 | 52.61 ± 15.86 |
| NO_2_, μg/m^3^ | 29.86 ± 10.87 | 29.23 ± 10.79 |
| O_3_, μg/m^3^ | 94.97 ± 7.36 | 95.33 ± 6.49 |

Data are shown as means ± standard deviations or numbers (percentages). The data in the excluded group were interpolated.

BMI body mass index, DBP diastolic blood pressure, NO_2_ nitrogen dioxide, O_3_ ozone, PM_1_ particulate matter with an aerodynamic diameter less than 1 μm, PM_2.5_ particulate matter with an aerodynamic diameter less than 2.5 μm, PM_10_ particulate matter with an aerodynamic diameter <10 μm, SBP systolic blood pressure.

**Table S16.** Baseline characteristics of included participants and those without lifestyle scores

|  | Without lifestyle score^a^  (n = 8047) | Included  (n = 7000) |
| --- | --- | --- |
| Age, years | 58.87 ± 10.34 | 58.43 ± 8.80 |
| Male | 3549 (52.80%) | 3247 (46.39%) |
| BMI, kg/m^2^ | 23.18 ± 4.03 | 23.49 ± 3.81 |
| Education |  |  |
| Primary school and below | 4132 (61.62%) | 5647 (70.47%) |
| Junior high school | 1501 (22.38%) | 1592 (19.87%) |
| High school and above | 1073 (16.00%) | 774 (9.66%) |
| Smoking | 2820 (41.95%) | 2678 (38.26%) |
| Alcohol consumption |  |  |
| More than once a month | 1811 (26.95%) | 1829 (26.13%) |
| Less than once a month | 573 (8.53%) | 575 (8.21%) |
| Never | 4335 (64.52%) | 4596 (65.66%) |
| SBP, mmHg | 130.55 ± 21.38 | 128.32 ± 20.84 |
| DBP, mmHg | 76.08 ± 11.99 | 74.98 ± 12.00 |
| Residence |  |  |
| Rural | 3673 (54.61%) | 4662 (66.60%) |
| Urban | 3053 (45.39%) | 2338 (33.40%) |
| Ambient air pollutants |  |  |
| PM_1_, μg/m^3^ | 40.45 ± 13.65 | 39.97 ± 13.80 |
| PM_10_, μg/m^3^ | 92.31 ± 28.24 | 93.44 ± 28.05 |
| PM_2.5_, μg/m^3^ | 52.45 ± 15.94 | 52.61 ± 15.86 |
| NO_2_, μg/m^3^ | 29.98 ± 10.93 | 29.23 ± 10.79 |
| O_3_, μg/m^3^ | 95.36 ± 7.16 | 95.33 ± 6.49 |
| Loss or death during follow-up period | 1321 (16.4%) | - |
| CVD^a^ | 882 (13.11%) | 1187 (16.9%) |

Data are shown as means ± standard deviations or numbers (percentages). The data in the group without lifestyle score were interpolated.

BMI body-mass index, DBP diastolic blood pressure, NO_2_ nitrogen dioxide, O_3_ ozone, PM_1_ particulate matter with an aerodynamic diameter less than 1 μm, PM_2.5_ particulate matter with an aerodynamic diameter less than 2.5 μm, PM_10_ particulate matter with an aerodynamic diameter <10 μm, SBP systolic blood pressure.

^a^The data were calculated after excluding those lost or who died during the follow-up period.
